# Supplementary material for: Chemoproteomic capture of RNA binding activity in living cells
Source: Nat Commun. 2023 Oct 7;14:6282. doi: 10.1038/s41467-023-41844-z (PMC10560261; doi:10.1038/s41467-023-41844-z)
Supplement: Supplementary file 1 — Supplementary Information [file 41467_2023_41844_MOESM1_ESM.pdf]

SUPPLEMENTARY INFORMATION

Chemoproteomic capture of RNA binding activity in living cells

Andrew J. Heindel[1], Jeffrey W. Brulet[2], Xiantao Wang[3], Michael W. Founds[2], Adam H. Libby[2,5], Dina L. Bai[2], Michael C. Lemke[1], David M. Leace[1], Thurl E. Harris[1], Markus Hafner[3] and Ku-Lung Hsu*[1,2,4,5]

[1]Department of Pharmacology, University of Virginia School of Medicine, Charlottesville, Virginia 22908, USA

[2]Department of Chemistry, University of Virginia, Charlottesville, Virginia 22904, USA

[3]RNA Molecular Biology Laboratory, National Institute of Arthritis and Musculoskeletal and Skin Disease, Bethesda, MD 20892, USA

[4]Department of Molecular Physiology and Biological Physics, University of Virginia, Charlottesville, Virginia 22908, USA

[5]University of Virginia Cancer Center, University of Virginia, Charlottesville, VA 22903, USA

*Author to whom correspondence should be addressed:
Present Address
Email: ken.hsu@austin.utexas.edu (K.-L.H.)
Department of Chemistry
University of Texas at Austin
100 East 24th Street
Austin, TX 78712
Phone: 512-232-1764

CONTENTS:

1. Supplementary Figures
2. Chemical Synthesis
3. Appendix
4. References

1. SUPPLEMENTARY FIGURES

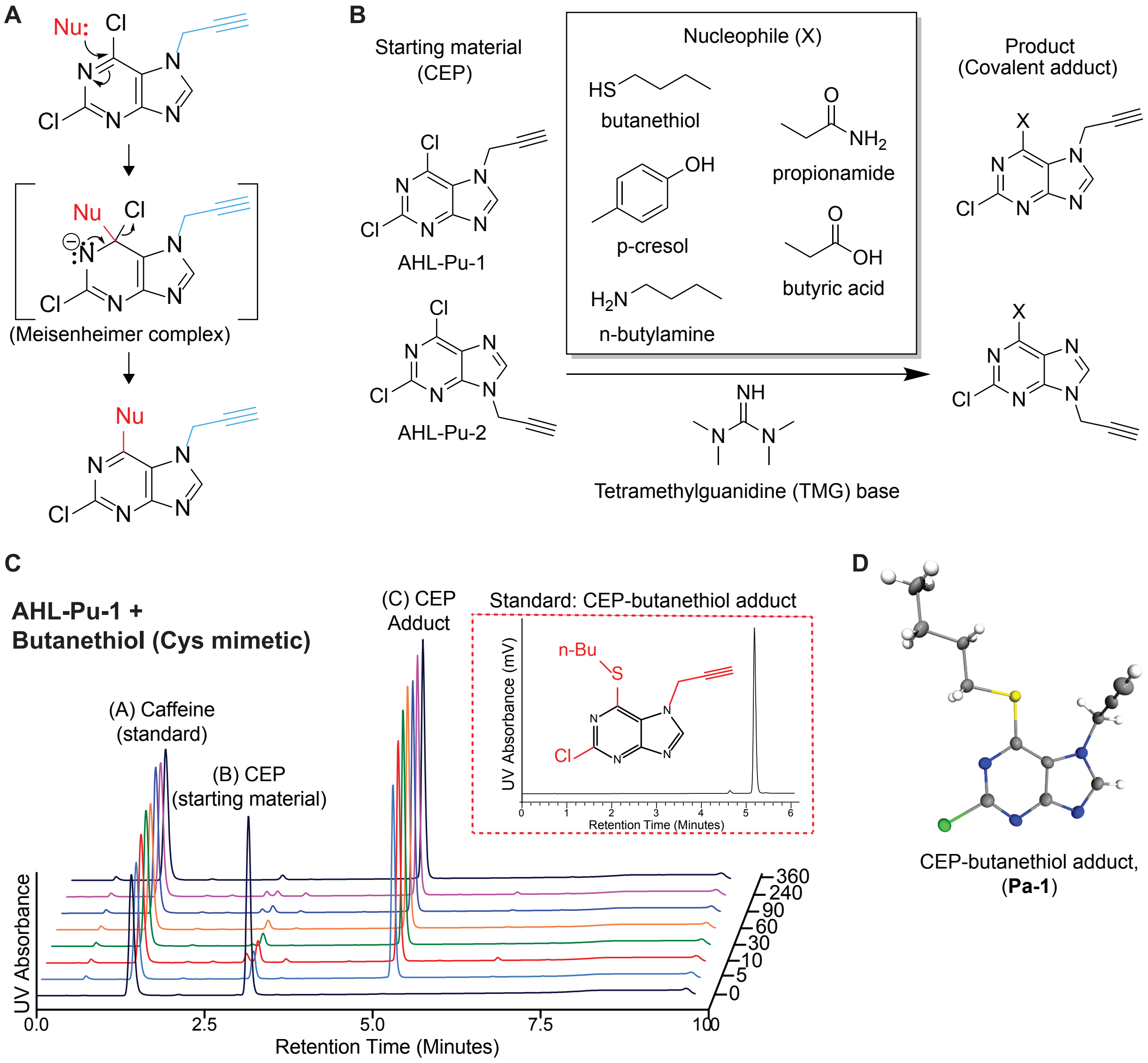

Supplementary Figure 1. **Reaction mechanism of purine-based probe molecules.** (A) The proposed nucleophilic aromatic substitution (S_NAr) reaction of CEPs with nucleophilic groups. (B) HPLC assay for measuring solution reactivity of CEPs. Time-dependent reactions were performed between nucleophiles (10.8 mM) with CEPs (AHL-Pu-1 or AHL-Pu-2; 9.8 mM). Tetramethylguanidine (TMG) was included as a base to facilitate covalent reaction. The following nucleophiles were chosen to mimic amino acid side chain groups: butanethiol (cysteine), *n*-butylamine (lysine), *p*-cresol (tyrosine), butyric acid (aspartate/glutamate), and propionamide (asparagine/glutamine). (C) Representative example of HPLC analysis of AHL-Pu-1 reaction to form the butanethiol-CEP adduct. Covalent reaction at C6 to form the AHL-Pu-1-butanethiol adduct was confirmed by retention times that matched those of the synthetic standard (Pa-1, D). See Supplementary Methods for additional details. Data shown are representative of three independent experiments (n=3).

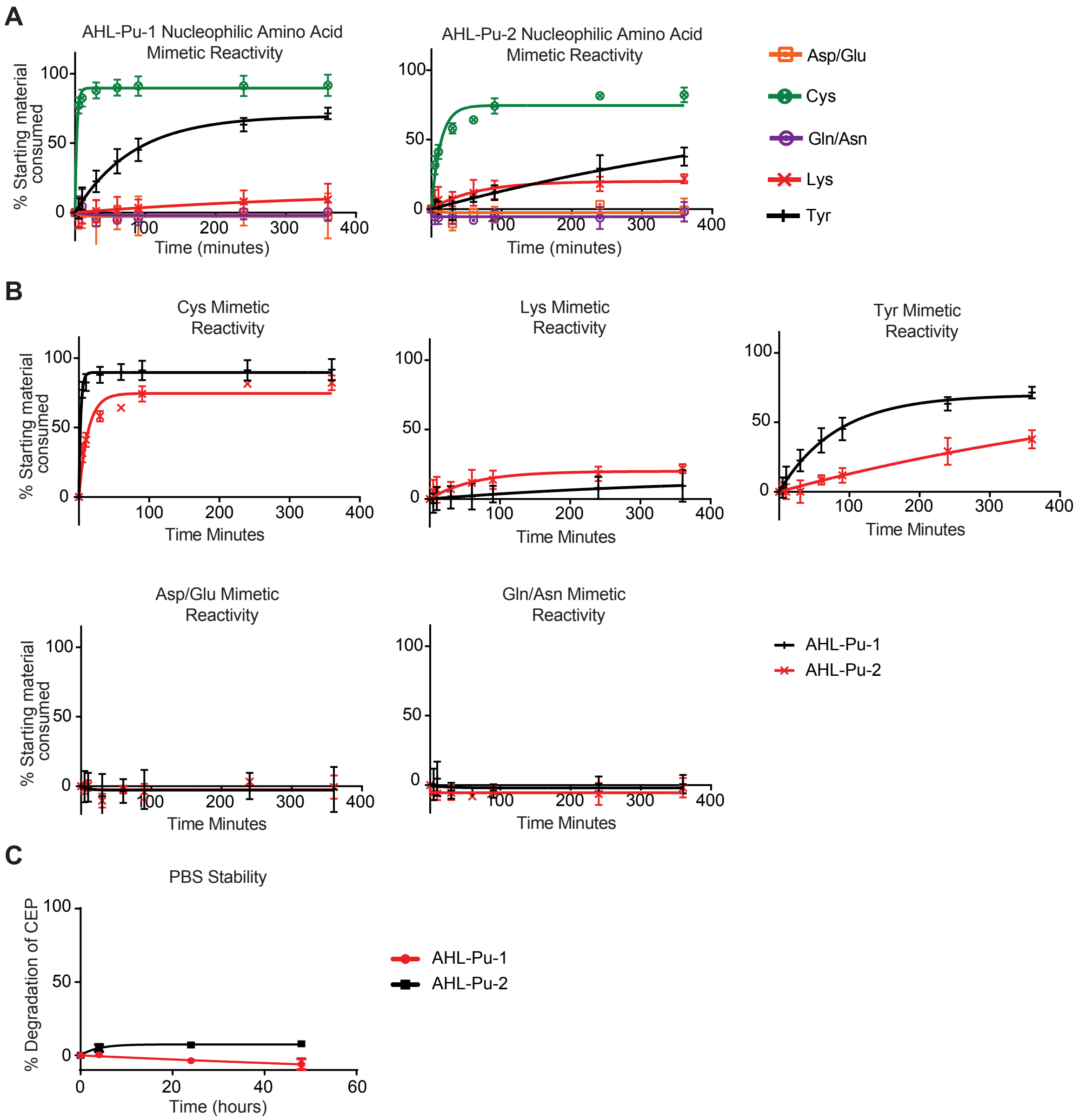

Supplementary Figure 2. **Comparison of AHL-Pu-1 and AHL-Pu-2 reactivity against nucleophiles in solution.** (A) Comparison of individual CEP reactivity against nucleophiles that mimic side chain functional groups of the indicated amino acid. See Supplementary Figure 1 for the set of nucleophiles tested. (B) Comparison of AHL-Pu-1 vs AHL-Pu-2 reactivity against nucleophiles in solution. HPLC assay was performed as depicted in Supplementary Figure 1 and described in Supplementary Methods. Data shown are representative of three independent experiments (n=3). (C) Stability of AHL-Pu-1 and AHL-Pu-2 in phosphate-buffered saline (PBS) buffer. Solutions of CEPs (9.8 mM) were prepared and HPLC analysis of these probes measured at the indicated time points. Negligible degradation, as determined by reduction of CEP signal, was observed after 48 hrs (2 days). See Supplementary Methods for additional details of the stability assay. Data shown are representative of three independent experiments (n=3).

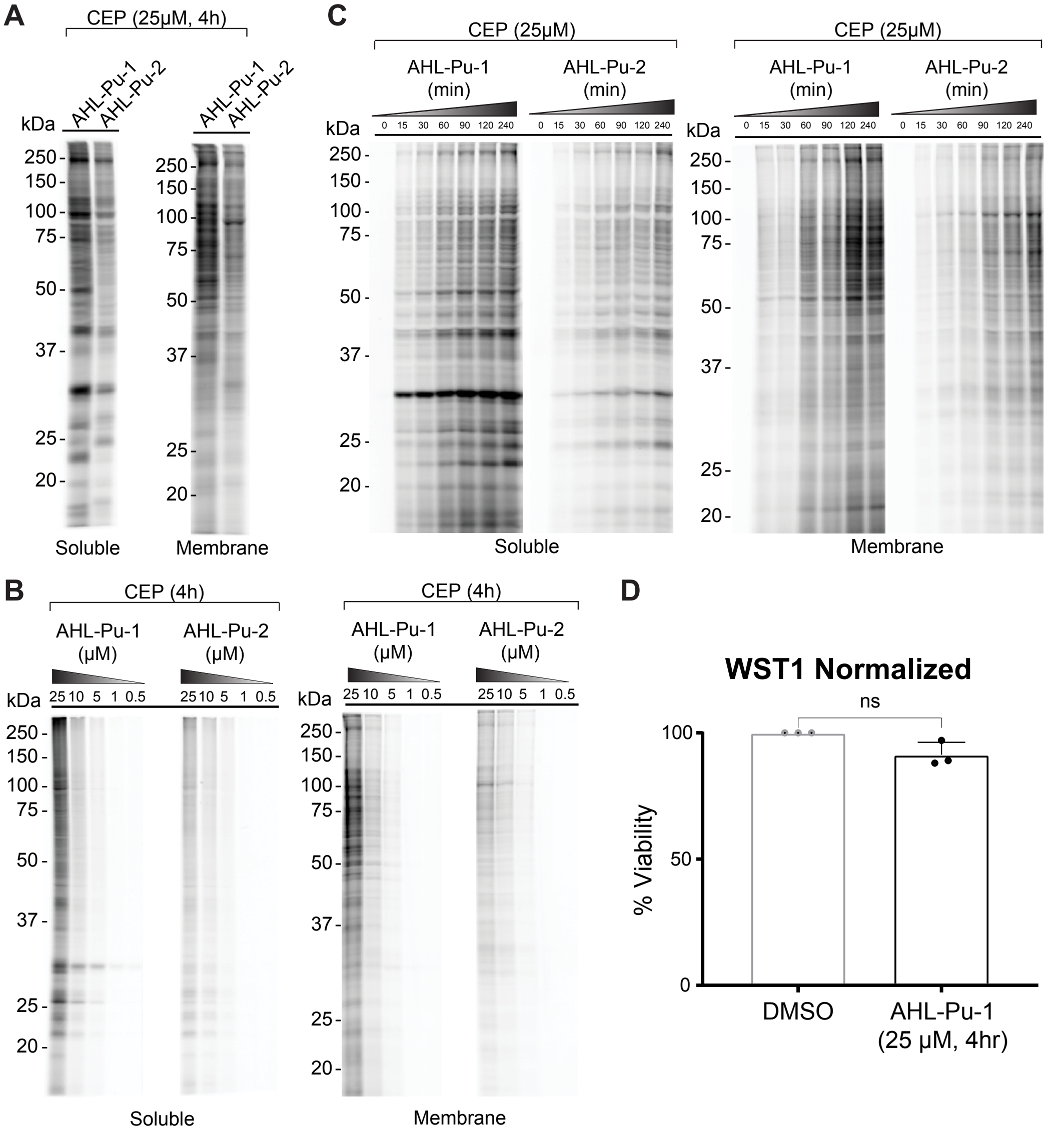

Supplementary Figure 3. **Live cell labeling using CEPs.** (A) Gel-based ABPP analysis of DM93 cells treated with CEP probes using optimized treatment conditions for LC-MS/MS quantitative chemical proteomics. DM93 cells were treated with 25 µM of AHL-Pu-1 or AHL-Pu-2 for 4 hr at 37 °C. After treatment, cells were lysed, probe-modified soluble (left panel; 2 mg/mL) and membrane proteomes (right panel; 2 mg/mL) subjected to CuAAC with rhodamine-azide followed by SDS-PAGE analysis and in-gel fluorescence scanning. (B) Concentration-dependent labeling of DM93 cells treated with CEP probes. DM93 cells were treated with indicated concentrations of AHL-Pu-1 or AHL-Pu-2 for 4 hr at 37 °C followed by gel-based ABPP analyses. (C) Time-dependent labeling of DM93 cells treated with CEP probes (25 µM of AHL-Pu-1 or AHL-Pu-2 for the indicated times at 37 °C) and subjected to gel-based ABPP analyses. (D) Cell viability of DM93 cells treated with AHL-Pu-1 (25 µM, 4 hr, 37 °C) as determined by the WST-1 assay for cell proliferation and viability. Cell viability was not statistically significantly different between DMSO vehicle and AHL-Pu-1 treated cells ($p = 0.4$). Statistical significance was determined using a Mann-Whitney test. Data shown are representative of n=3 biologically independent experiments.

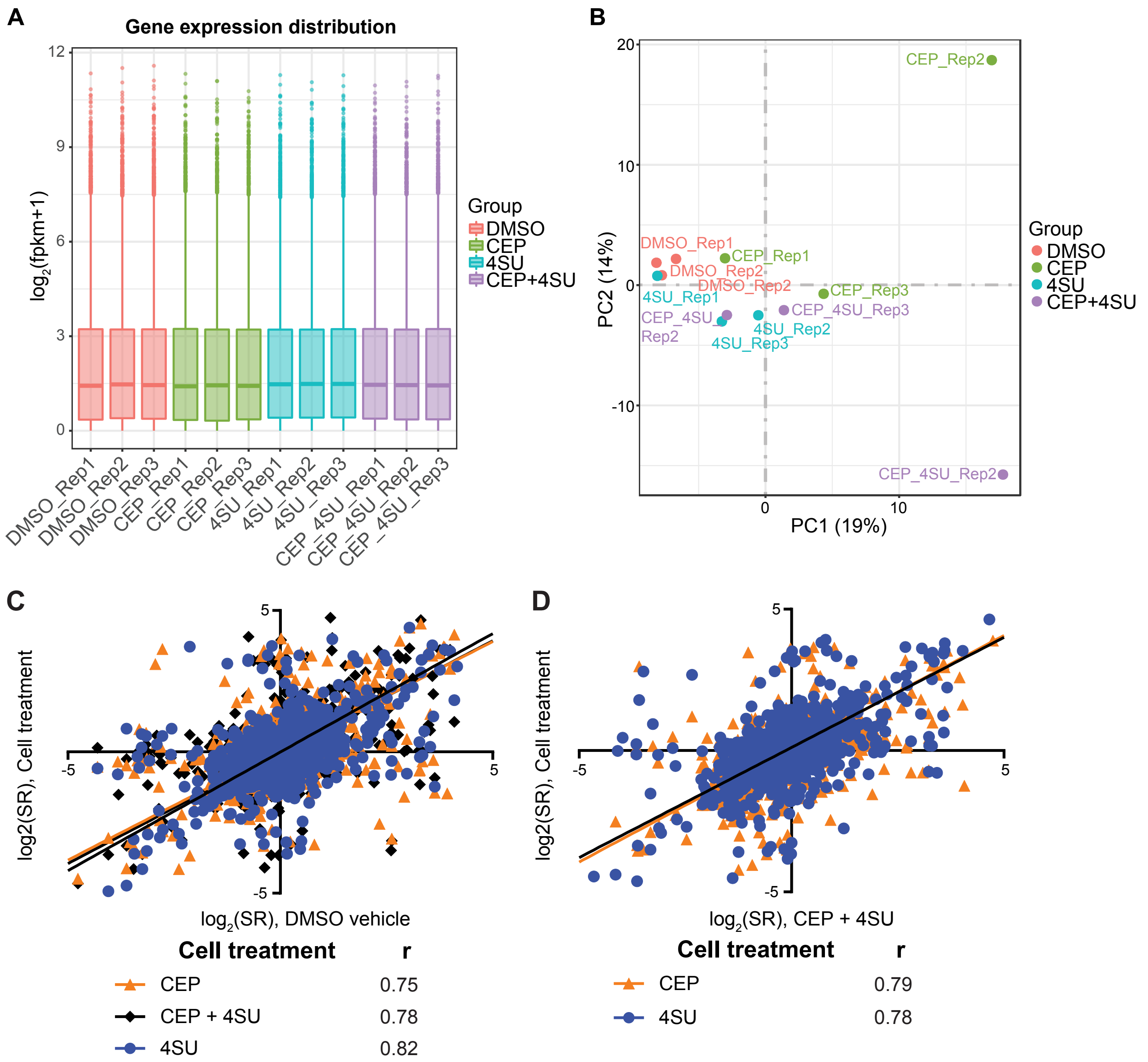

Supplementary Figure 4. **Experimental conditions used for PACCE in HEK293T cells do not generally alter the transcriptome or proteome.** (A) Normalized expression values (fragments per kilobase of transcript per million mapped reads [FPKM]) as determined by paired-end RNA-Seq. Respective samples were treated with either 4SU (100 µM, 16 hr), CEP (25 µM, 1 hr), or both at 37 °C. Cells were processed using a PureLink RNA Mini Kit. (B) Principal component (PC) analysis results derived from RNA-Seq samples. The X and Y axes indicate PC1 and PC2, which explain 14% and 19% of the total variation, respectively. The correlation scatter plot of SR values ($\log_2$) of different cell treatments compared to DMSO (C) or PACCE condition (CEP+4SU; D) to assess proteomic alterations. Peaks defined by $\log_2$(L/H ratios) >5 were set to 5. Data shown are representative of n=3 biologically independent experiments.

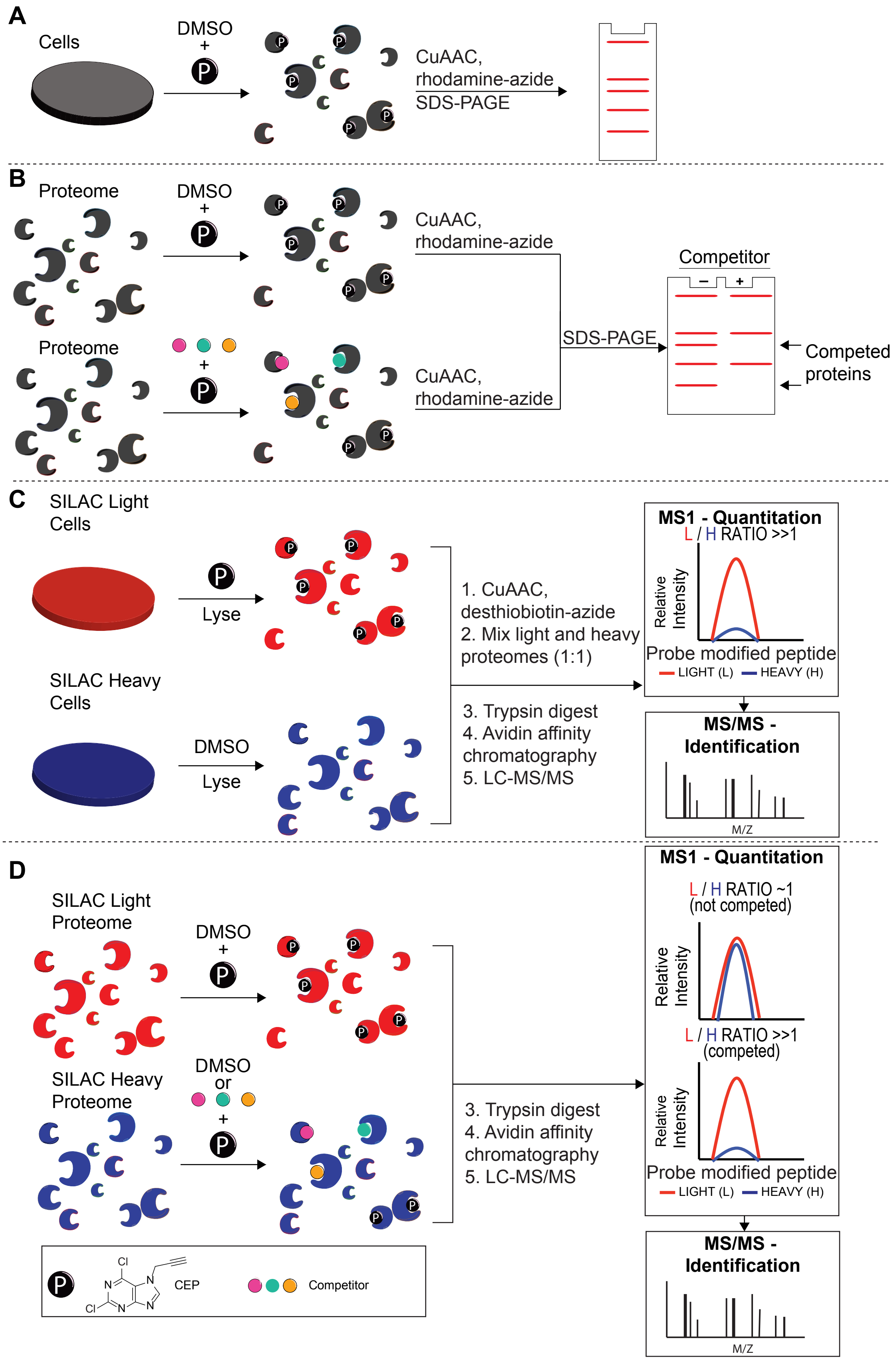
A
Cells
DMSO
+
P
CuAAC,
rhodamine-azide
SDS-PAGE
B
Proteome
DMSO
+
P
CuAAC,
rhodamine-azide
Competitor
– +
SDS-PAGE
Competed
proteins
Proteome
+
P
CuAAC,
rhodamine-azide
C
SILAC Light
Cells
P
Lyse
1. CuAAC,
desthiobiotin-azide
2. Mix light and heavy
proteomes (1:1)
MS1 - Quantitation
L / H RATIO >>1
Relative
Intensity
Probe modified peptide
LIGHT (L)
HEAVY (H)
SILAC Heavy
Cells
DMSO
Lyse
3. Trypsin digest
4. Avidin affinity
chromatography
5. LC-MS/MS
MS/MS -
Identification
M/Z
D
SILAC Light
Proteome
DMSO
+
P
MS1 - Quantitation
L / H RATIO ~1
(not competed)
Relative
Intensity
L / H RATIO >>1
(competed)
SILAC Heavy
Proteome
DMSO
or
+
P
3. Trypsin digest
4. Avidin affinity
chromatography
5. LC-MS/MS
Probe modified peptide
LIGHT (L)
HEAVY (H)
MS/MS -
Identification
M/Z
CEP
Competitor

Supplementary Figure 5. Experimental workflow for CEP-mediated chemical proteomics. (A) Gel based profiling studies (*in situ*). Cells are treated with either CEP or DMSO. Cells are lysed and conjugated to a fluorescent tag by CuAAC and analyzed with SDS-PAGE. Schematic corresponds to probe-labeling SDS-PAGE experiments in Supplementary Figure 4. (B) Gel-based competition studies. Proteome derived from lysed cells is pretreated with either CEP + DMSO or CEP + inhibitor. Next, samples are conjugated to a fluorescent dye via CuAAC and analyzed with SDS-PAGE. Loss of fluorescent signal indicates competition. Figure corresponds to probe-labeling SDS-PAGE experiments in Supplementary Figure 7. (C) Non-competitive workflow to identify proteins and corresponding binding sites that are enriched from CEP labeling of proteins in cells. For SILAC workflows, proteomes are derived from cells cultured in SILAC media supplemented with either "light" ^{12}C, ^{14}N- (denoted in red) or "heavy" ^{13}C, ^{15}N-labeled lysine and arginine (denoted in blue). To identify CEP-enriched proteins, light and heavy cells are treated with CEP probe (25 μM, 4 hr, 37 °C) or DMSO vehicle, respectively. Afterwards, cells are lysed followed by CuAAC conjugation of desthiobiotin-azide, avidin affinity chromatography, and LC-MS/MS analysis. The resulting SILAC ratios (SR) are quantified using the area under the curve of MS1 EICs. CEP-enriched proteins are identified using probe-modified peptides that meet quality control criteria and show a substantial increase in peptide abundance in CEP probe-treated compared with vehicle control samples (SR >>1). Schematic corresponds to Figure 1B-D. (D) Competitive workflow to evaluate inhibitor activity in proteomes. Cell proteomes derived from either light- or heavy-labeled cells were co-treated with DMSO vehicle or nitrogenous base (0.025-25 mM, 30 min, 37 °C), respectively, and CEP probe (25 μM). Non-competed sites are expected to show equivalent probe labeling intensity in vehicle (L)- and fragment (H)-treated conditions (SR~1). Nitrogenous base-competed sites are identified by probe-modified peptides showing a substantial reduction in peptide abundance (due to competition of CEP labeling) in nitrogenous base- compared with vehicle-treated control samples (SR >>1). This workflow was used for LC-MS/MS studies shown in Supplementary Figure 7. Additional details of the chemoproteomic assays can be found in Supplementary Methods.

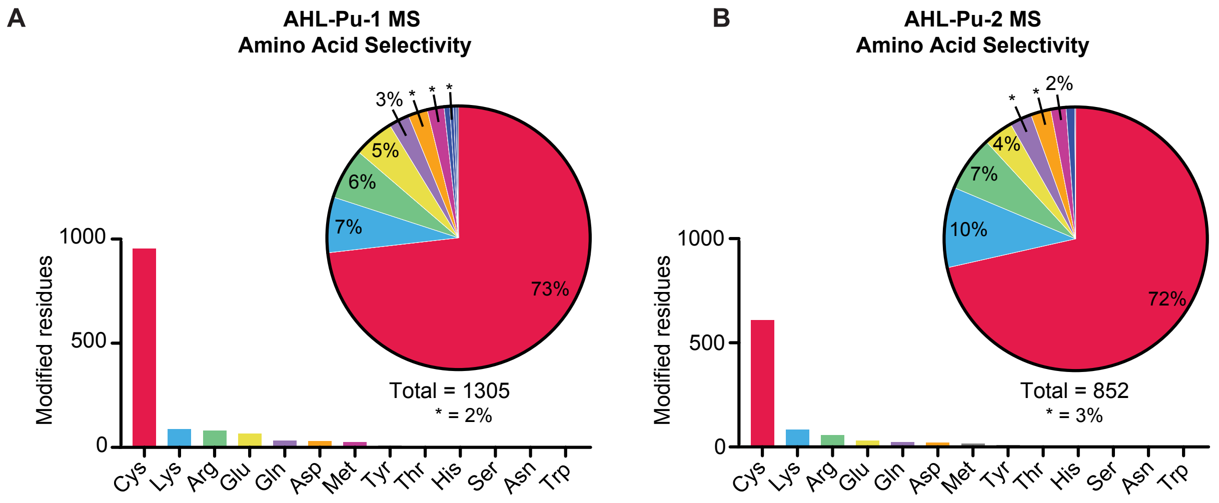

Supplementary Figure 6. **Amino acid preference of CEPs in live cell environment.** Distribution of CEP modifications on nucleophilic amino acid residues from probe-modified peptides of detected proteins (soluble proteomes) from quantitative chemoproteomic analyses of CEP-treated DM93 cells. Data shown are high confidence sites (Byonic score $\geq$ 600) for AHL-Pu-1 (A) and AHL-Pu-2 (B) and representative of n=3 biologically independent experiments.

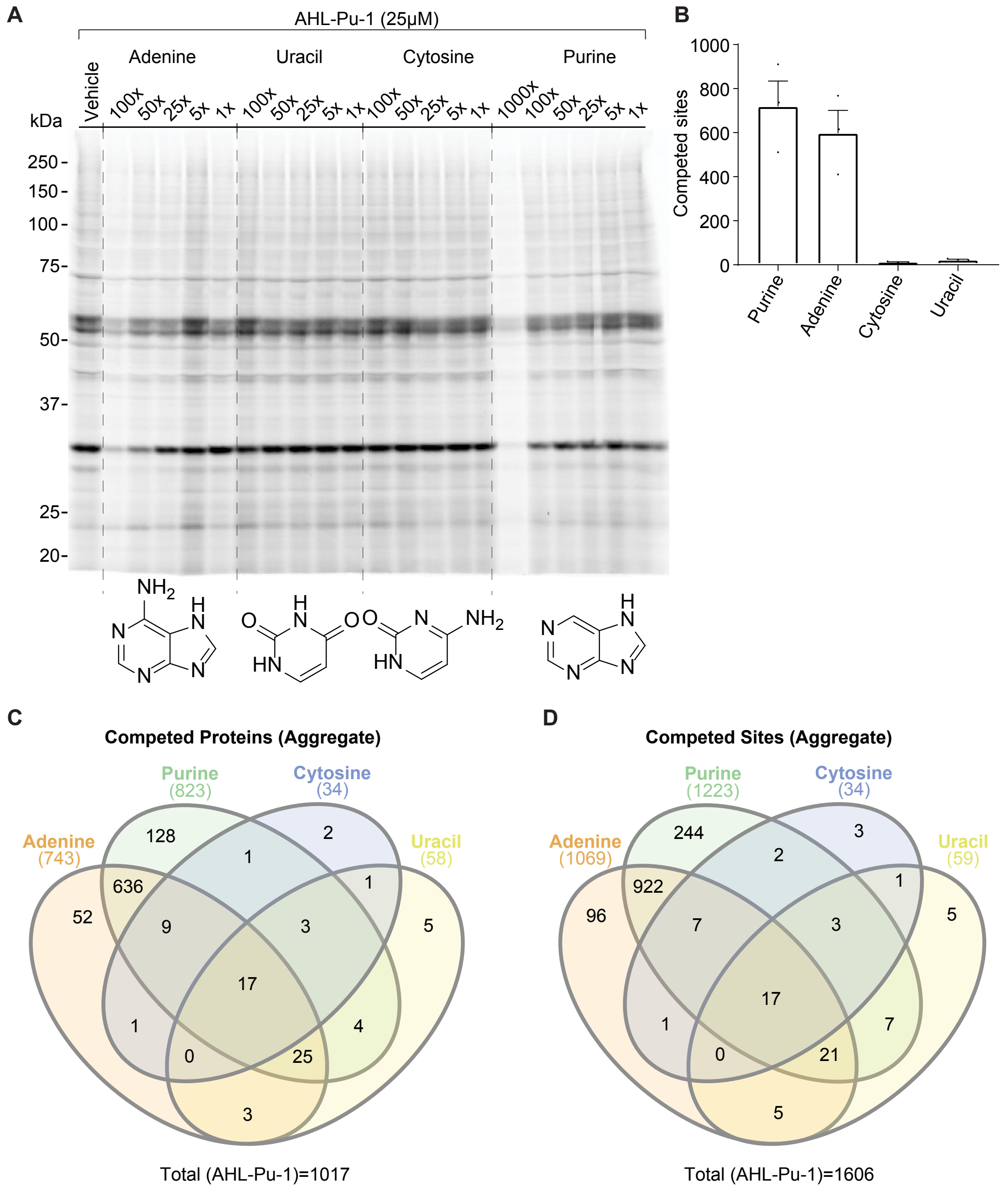

Supplementary Figure 7. **CEP competition against purine and pyrimidine bases.** The protein-binding profiles of AHL-Pu-1-labeled HEK293T proteomes are competed by pretreatment of purines but not pyrimidines *in vitro* as determined by gel (A) and quantitative chemical proteomics (B). These studies provide evidence that AHL-Pu-1 covalent binding activity is dependent on purine recognition. Quantitative chemical proteomics showed that CEP-modified proteins (C) and sites (D) are largely competed with purine (25 mM) and adenine (2.5 mM) but not uracil (2.5 mM) or cytosine (2.5 mM). Proteomes were co-treated with nitrogenous bases at the indicated concentrations for 30 min at 37 °C and AHL-Pu-1 (25 µM, 30 min, 37 °C). Data shown are representative of n=3 biologically independent experiments. See Supplementary Figure 5 and Supplementary Methods for additional details.

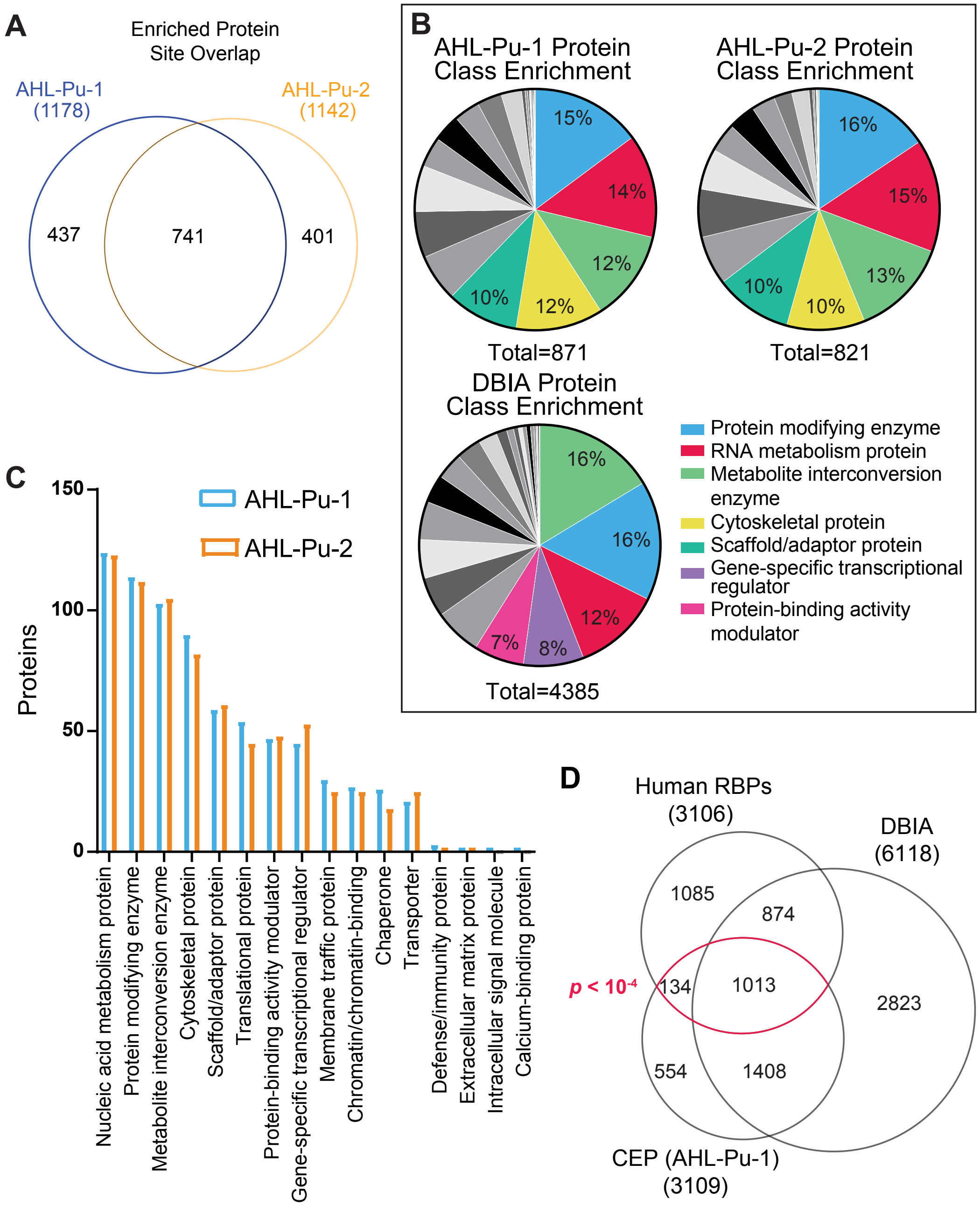

Supplementary Figure 8. **Structure-activity relationship (SAR) of CEP analogs. DM93 cells were treated with AHL-Pu-1 (N_7 alkyne handle) or AHL-Pu-2 (N_9 alkyne handle) at 25 µM for 4 hr (37 °C).** (A) Venn diagram of overlapping sites between CEP analogs. Functional protein classes enriched in CEP-treated DM93 cells as determined by Gene Ontology[1,2], highlighting AHL-Pu-1 and AHL-Pu-2 modify an equivalent array of protein classes using panther classification (B) and GO functions (C). A full list of functional protein classes from Panther classifications can be found in Supplementary Data 1. Sites with specific enrichment (SR > 5) were quantified. Data shown are representative of n=3 biologically independent experiments. (D) Overlap of CEP and DBIA datasets with human annotated RBPs[3]. Overlap between CEP-enriched proteins and human annotated RBPs was statistically significant as determined by hypergeometric distribution ($p = 4.09 \times 10^{-5}$). See Supplementary Methods for additional information.

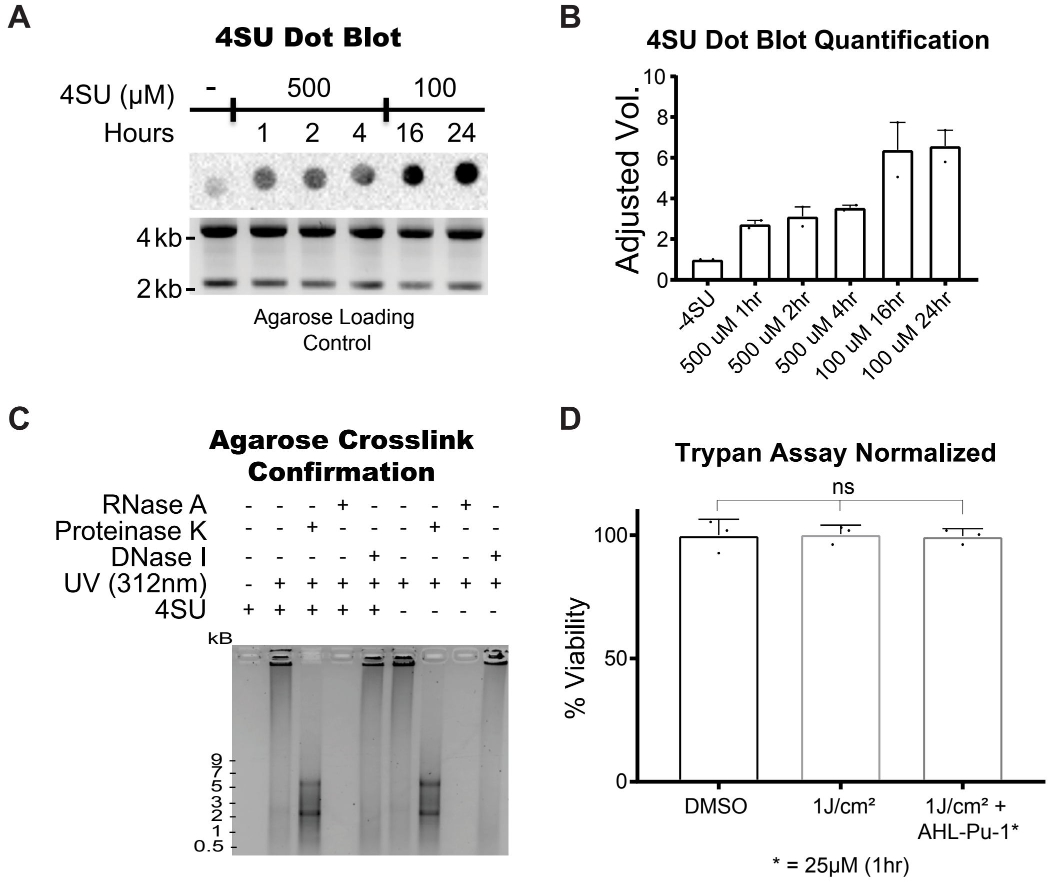

Supplementary Figure 9. **Identifying optimal conditions for 4SU metabolic labeling of cellular RNA in HEK293T cells.** Optimizing non-toxic conditions for metabolic incorporation of 4SU into cellular RNA using RNA dot blots (A, B) and agarose gel analyses following published methods[4]. (C) UV irradiation at 312 nm to crosslink 4SU-labeled cellular RNA to proteins was confirmed by agarose gel-shift assays. Photocrosslinking of native RNA to proteins was also observed at this wavelength. (D) Photocrosslinking using optimized 4SU conditions (100 µM, 16 hr) did not result in overt toxicity to cells. Data shown are representative of n=3 biologically independent experiments. Details on statistical tests used can be found in Supplementary Methods.

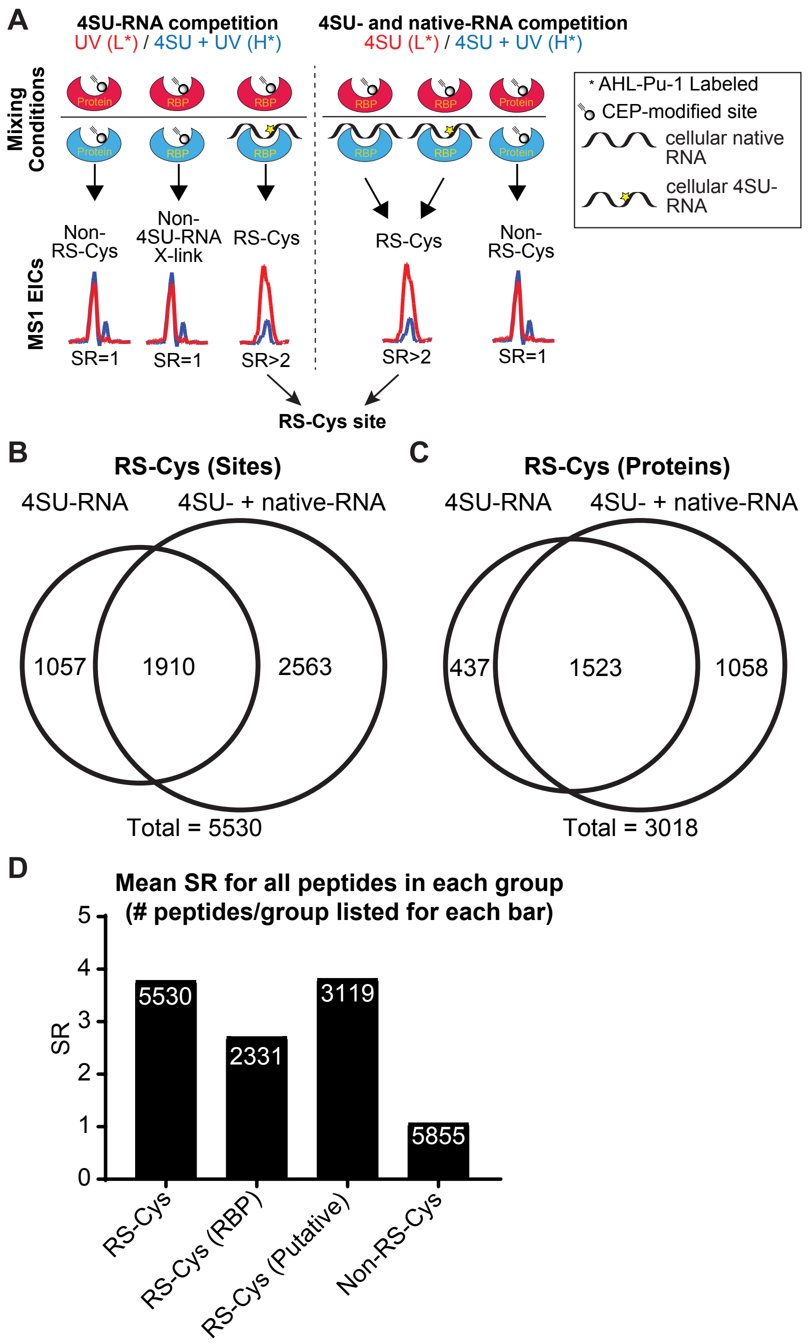

Supplementary Figure 10. **PACCE conditions for quantifying RNA-sensitive cysteine (RS-Cys) sites.** (A) UV crosslinking of RNA to proteins protects cysteines located in or proximal to RNA-binding sites from CEP labeling. PACCE captures RS-Cys sites competed (SR >2) by crosslinking (i) 4SU-RNA and (ii) 4SU- and native-RNA. RS-Cys sites (5,530, B) and proteins (3,018, C) were determined by the aggregate of all sites that show sensitivity to RNA crosslinking competition from PACCE *in situ* (live cell labeling using CEP probe). (D) Average SR value for peptides found in respective groups. The number of reported sites per group is highlighted. Data shown are representative of n=3 independent experiments from PACCE studies in HEK and DM93 cells.

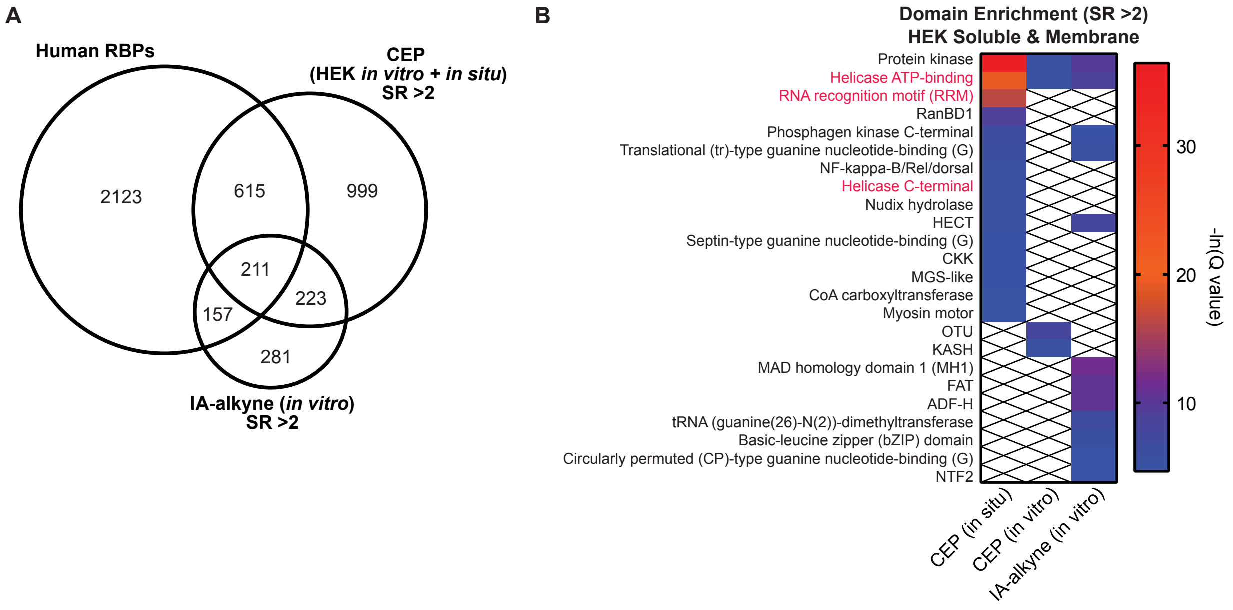

Supplementary Figure 11. **Comparison of PACCE using CEP (*in vitro* and *in situ*) and IA-alkyne (*in vitro*) probe in HEK293T cells and proteomes.** (A) Venn diagram of overlapping sites between IA-alkyne and CEP. (B) Domain enrichment analysis of CEP- and IA-alkyne modified sites identified from HEK soluble and membrane fractions. Domain enrichment analyses were performed as previously described[5]. Data shown are representative of n=3 biologically independent experiments.

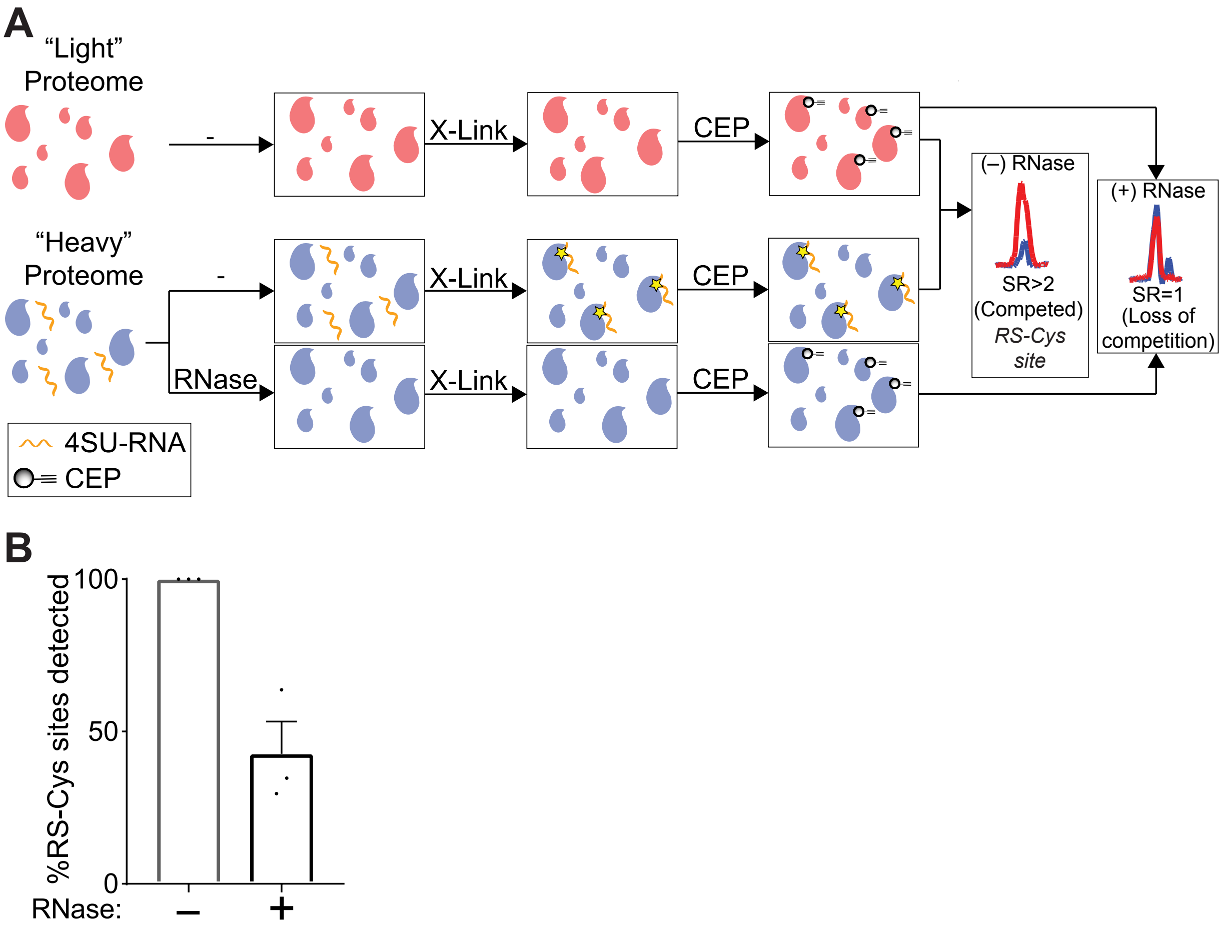

Supplementary Figure 12. **RNase treatment reduces RS-Cys detection by PACCE in HEK293T cell proteomes.** (A) Workflow for evaluating effects of RNase treatment on RS-Cys detection by PACCE. SILAC light and heavy cells are cultured in the absence or presence of 4SU-RNA, respectively. Cells were lysed and proteomes exposed to UV irradiation followed by CEP probe labeling and quantitative chemical proteomics to identify RS-Cys sites (SR >2). The role of crosslinked 4SU-RNA in protecting RNA-binding sites from CEP labeling was verified by addition of RNase to proteomes prior to UV irradiation. (B) Quantitation of RNase treatments in PACCE studies. RNase treatment of lysates resulted in a 60% reduction in the number of RS-Cys sites detected [a total of 934 and 317 RS-Cys sites in (-)RNase and (+)RNase sample groups, respectively]. Data shown are mean ± SEM for n = 3 biologically independent experiments.

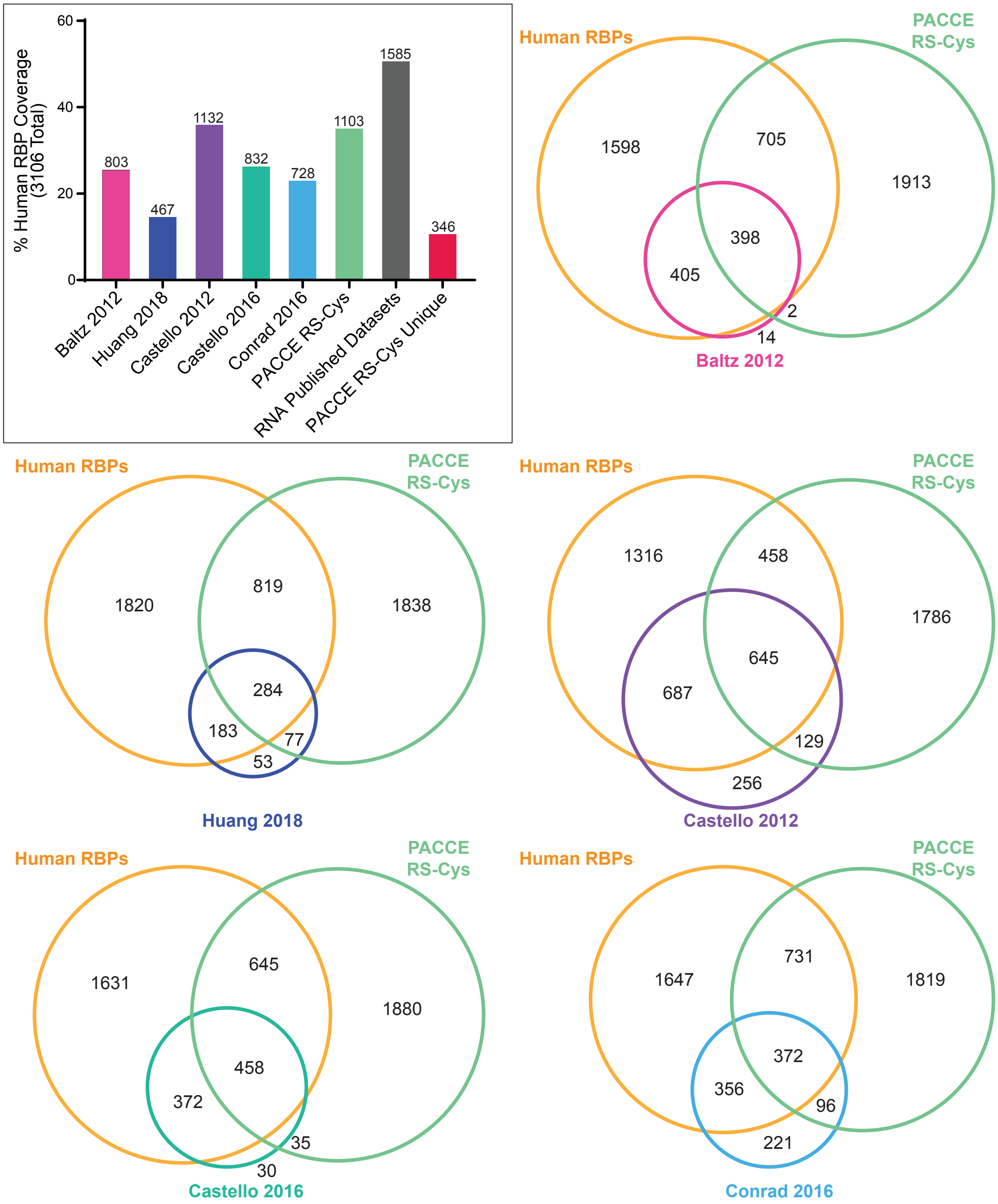

Supplementary Figure 13. **RS-Cys-containing proteins compared with RBPs detected by RIC methods and the curated human RBP dataset[3,6-9]**. Inset depicts coverage of Human Annotated RBPs by CEP and RIC methods. PACCE detects an additional 346 RBPs not captured by the 5 existing RIC methods.

A

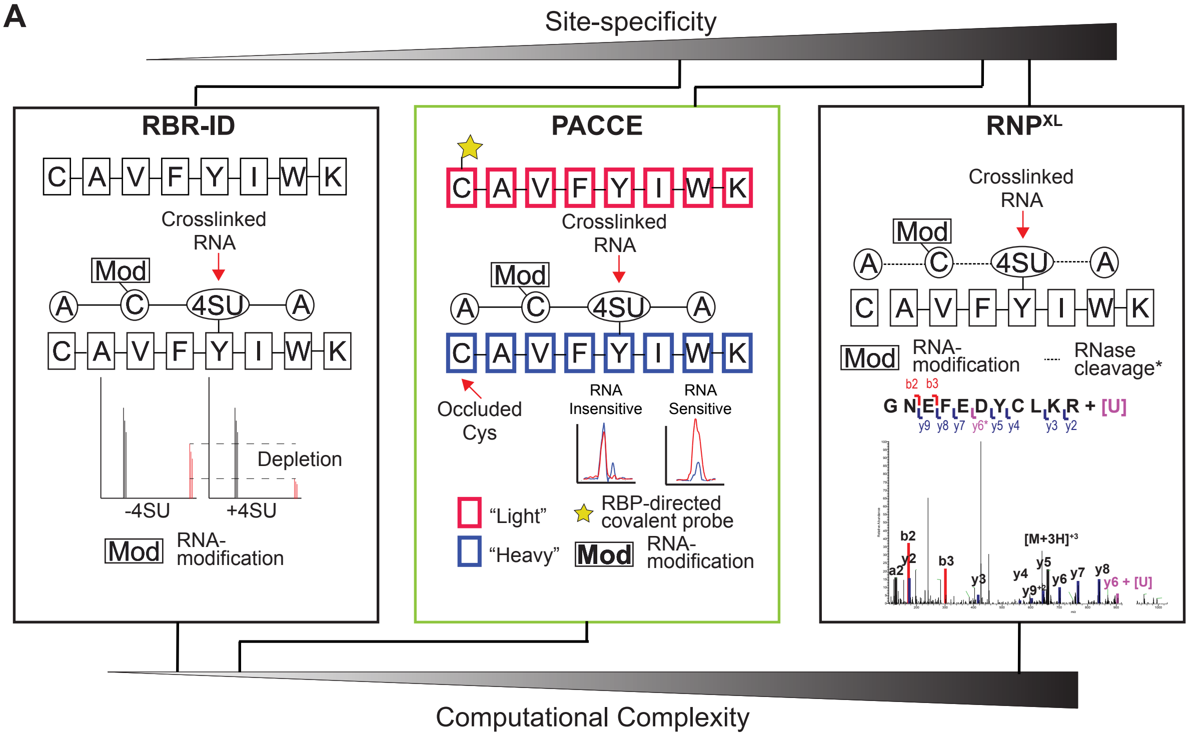

B

Comparison of PACCE with RIC methods

RBR-ID	PACCE	RNP^{XL}	
No	No	Yes	RNA X-link ID
No	Yes	Yes	Binding site ID
No	No	Yes	RNA modification required (polyA)
No	Yes	No	Small molecule screening
Yes	Yes	No	Compatible with standard computational workflows for proteomics

Supplementary Figure 14. Comparison of PACCE versus other proteomic methods for RBP analysis. (A) Descriptions of mass spectrometry based RBP identification methods. (B) Table of key advantages and disadvantages for each respective method compared.

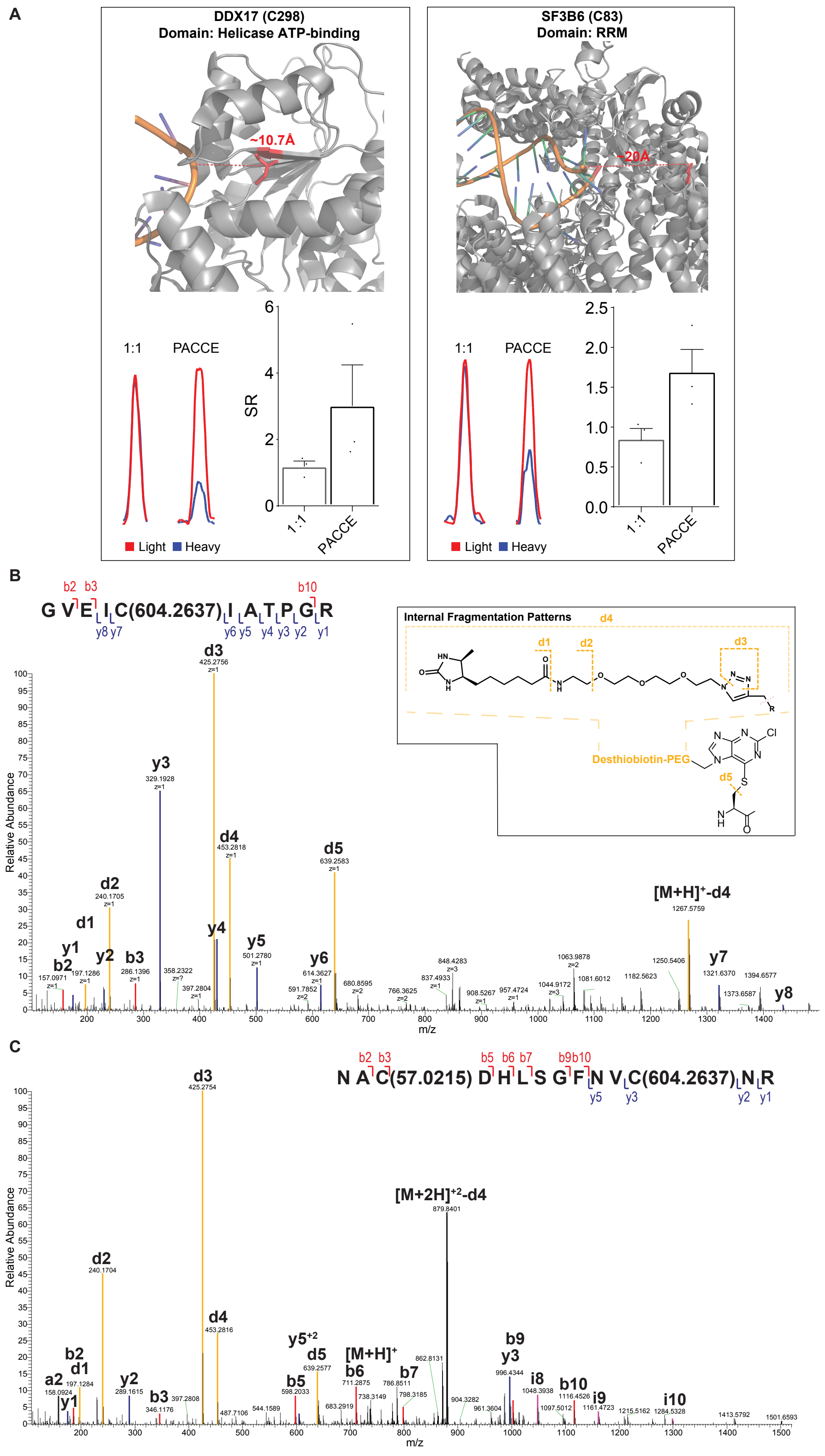
A
DDX17 (C298)
Domain: Helicase ATP-binding
~10.7Å
1:1
PACCE
SR
Light
Heavy
SF3B6 (C83)
Domain: RRM
~20Å
B
G V E I C(604.2637) I A T P G R
b2 b3 b10
y8 y7 y6 y5 y4 y3 y2 y1
Internal Fragmentation Patterns
d1 d2 d3 d4 d5
Desthiobiotin-PEG
Relative Abundance
m/z
[M+H]+-d4
C
N A C(57.0215) D H L S G F N V C(604.2637) N R
b2 b3 b5 b6 b7 b9b10
y5 y3 y2 y1
[M+2H]+2-d4
[M+H]+
y5+2
i8 i9 i10

Supplementary Figure 15. **Identification of known RBPs using PACCE.** (A) Location and distance of RS-Cys sites to RNA in RBP-RNA structures: DDX17, C298 (6UV2, X-ray); SF3B6, C83 (7Q4O, X-ray). Cys residues reside in canonical RBDs, including RRM and Helicase-ATP binding domains. DDX17 probe-modified peptide is shared with DDX5. Distances were calculated using Pymol. Details on statistical analysis can be found in Supplementary Methods. Data shown are mean $\pm$ SEM for n=3 biologically independent replicates for each treatment condition. *$p \leq 0.05$. (B) Mass Spectrometry analysis of RNA sensitive, AHL-Pu-1-modified tryptic peptide on DDX17/DDX5 (C298/C221). Covalent addition of AHL-Pu-1 onto the Cys residues results in a modified Cys (C*) with a mass addition of +604.2631 Da. In addition to standard b- and y-fragment ions, internal fragment ions due to fragmentation of AHL-Pu-1 probe are highlighted in teal or pink, respectively. Internal fragmentation of y- and b-fragment ions that contain a portion of the probe are denoted by iX and fX annotations, respectively. Yellow peaks denote desthiobiotin fragments. Data shown are representative of 3 biologically independent replicates. The average SR value across biological replicates can be found in Supplementary Data 4. (C) Modified sequence and MS2 fragment ion annotation of the SF3B6 C83 site.

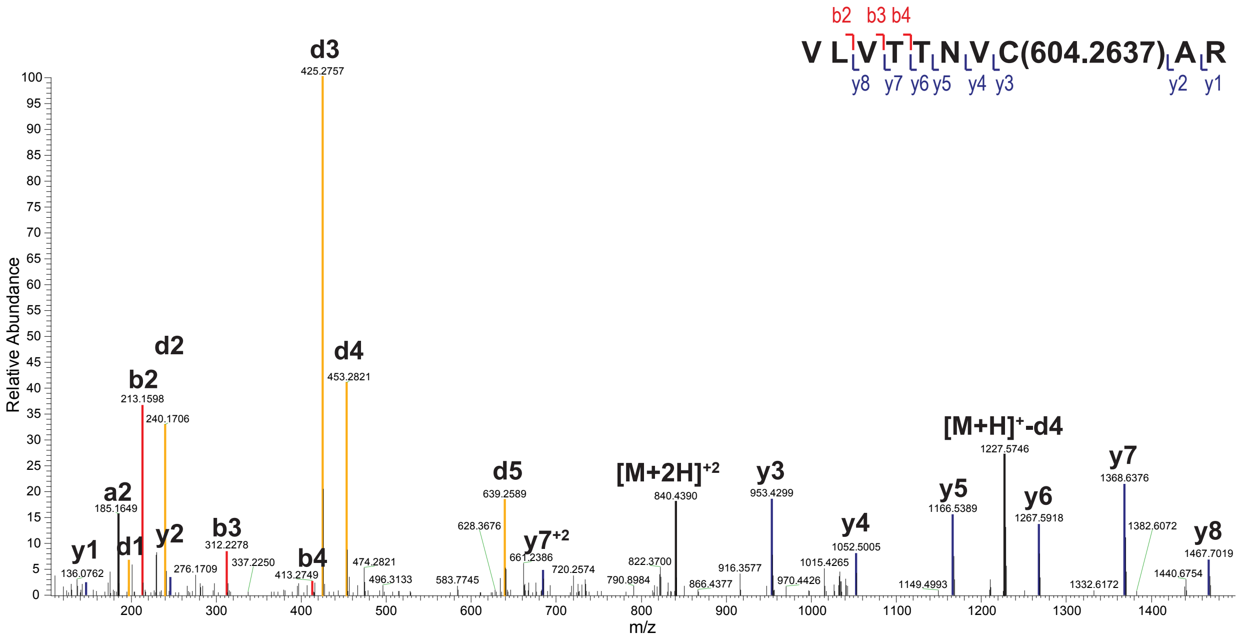

Supplementary Figure 16. Mass Spectrometry analysis of the RS-Cys site on DDX19B (C393). Modified sequence and MS2 fragment ion annotation of VLVTTNVC*AR peptide from DDX19B. This Cys site is also found in DDX19A (C392). See Supplementary Figure 15 for details on the annotation and interpretation of MS2 data.

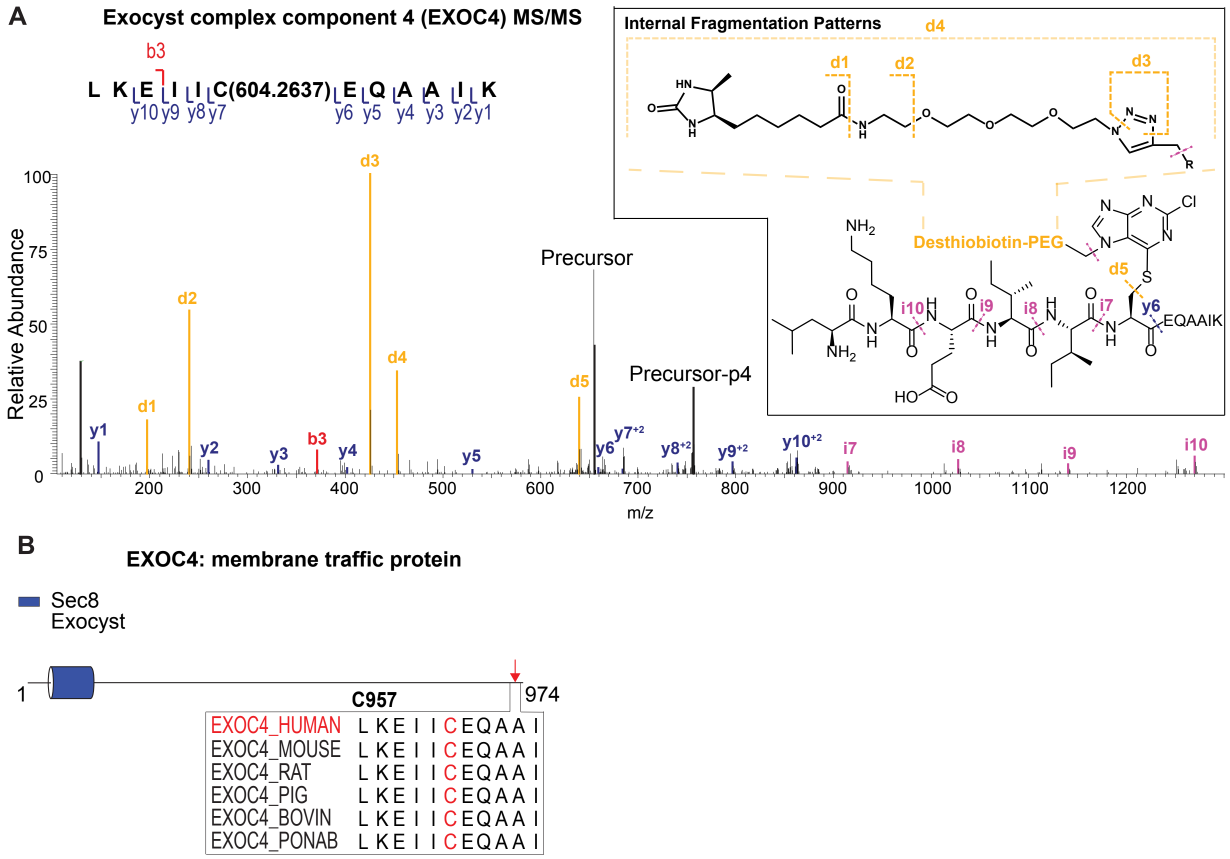

Supplementary Figure 17. **Mass Spectrometry analysis of the RS-Cys site on EXOC4 (C957).** (A) MS2 fragment ion annotation of LKEIIC*EQAAIK peptide from EXOC4. Inset contains a schematic of fragments from probe and internal fragmentation. (B) EXOC4 domains. The RS-Cys site is highlight with a red arrow. See Supplementary Figure 15 for details on the annotation and interpretation MS2 data.

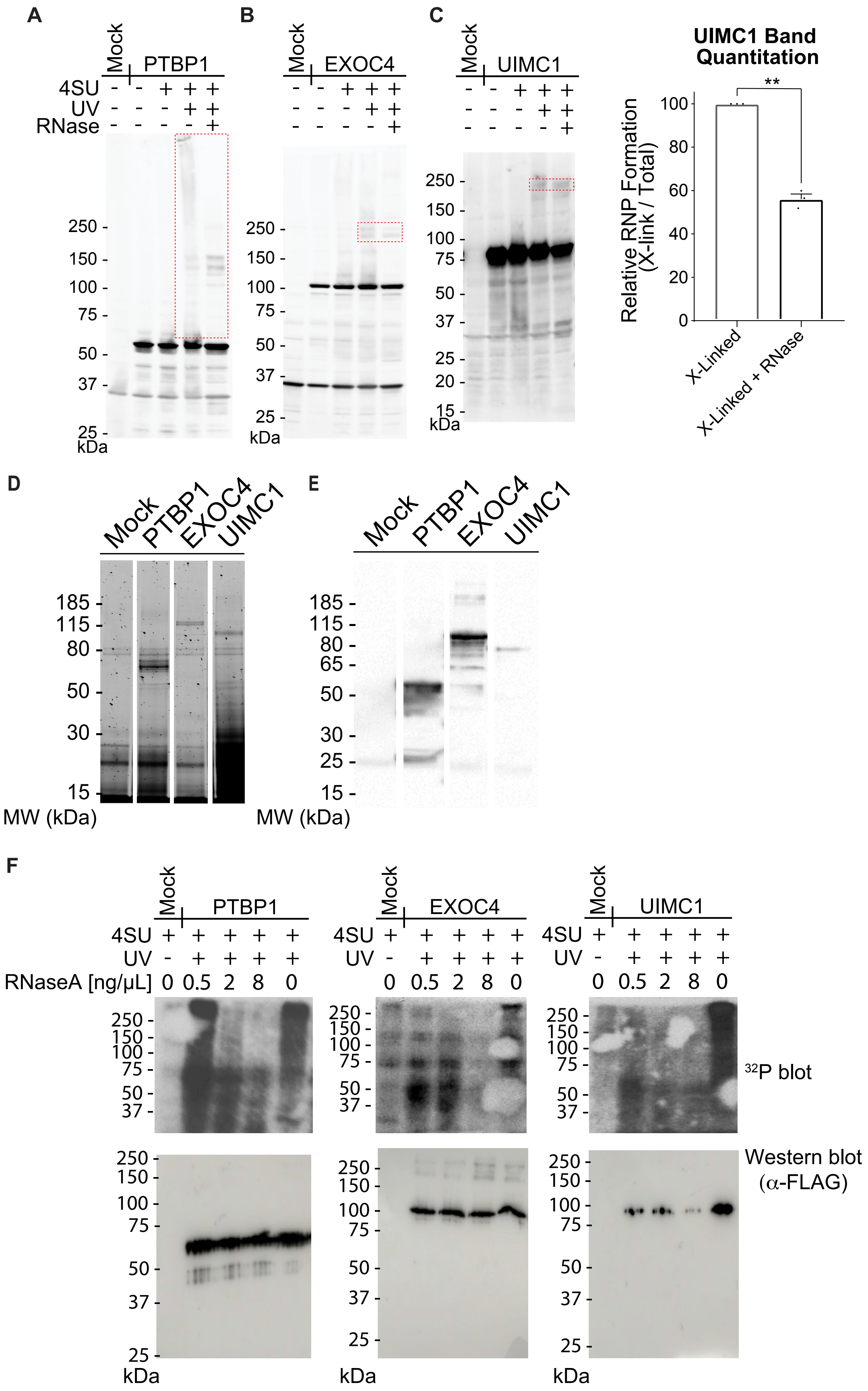
A
B
C
D
E
F
Mock
PTBP1
EXOC4
UIMC1
4SU
UV
RNase
kDa
UIMC1 Band Quantitation
Relative RNP Formation (X-link / Total)
X-Linked
X-Linked + RNase
MW (kDa)
RNaseA [ng/µL]
32P blot
Western blot (α-FLAG)

Supplementary Figure 18. **Validation of novel RBP activity in PACCE-identified proteins.** Western blot analyses showing RNP formation upon UV crosslinking in HEK293T cells expressing recombinant proteins of PTBP1 (A), EXOC4 (B), and UIMC1 (C). RNA crosslinking in cells forms higher molecular weight RNA-protein complexes that are detected by reduced migration using SDS-PAGE gels and exhibit sensitivity to RNase treatment (highlighted in red box). Quantification showed significant reductions in UIMC1 band signals from RNase treatment. Data shown are mean + SEM for n=3 biologically independent replicates. **p < 0.01. Full length fluorescent gel (D) and western blot (E) of fPAR-CLIP evaluation of FLAG-PTBP1, -EXOC4, and -UIMC1. (F) Cellular lysates recombinantly expressing proteins of interest from UV-treated or control cells were subjected to concentration-dependent RNaseA treatments. Proteins were immunoprecipitated followed by P32 radioactive labeling (top) and western blotting (bottom). Proteins were immunoprecipitated followed by radioactive labeling (32P) of RNA 5′ ends with T4 polynucleotide kinase and imaging of autoradiographic film. Western blotting was performed to confirm recombinant protein expression.

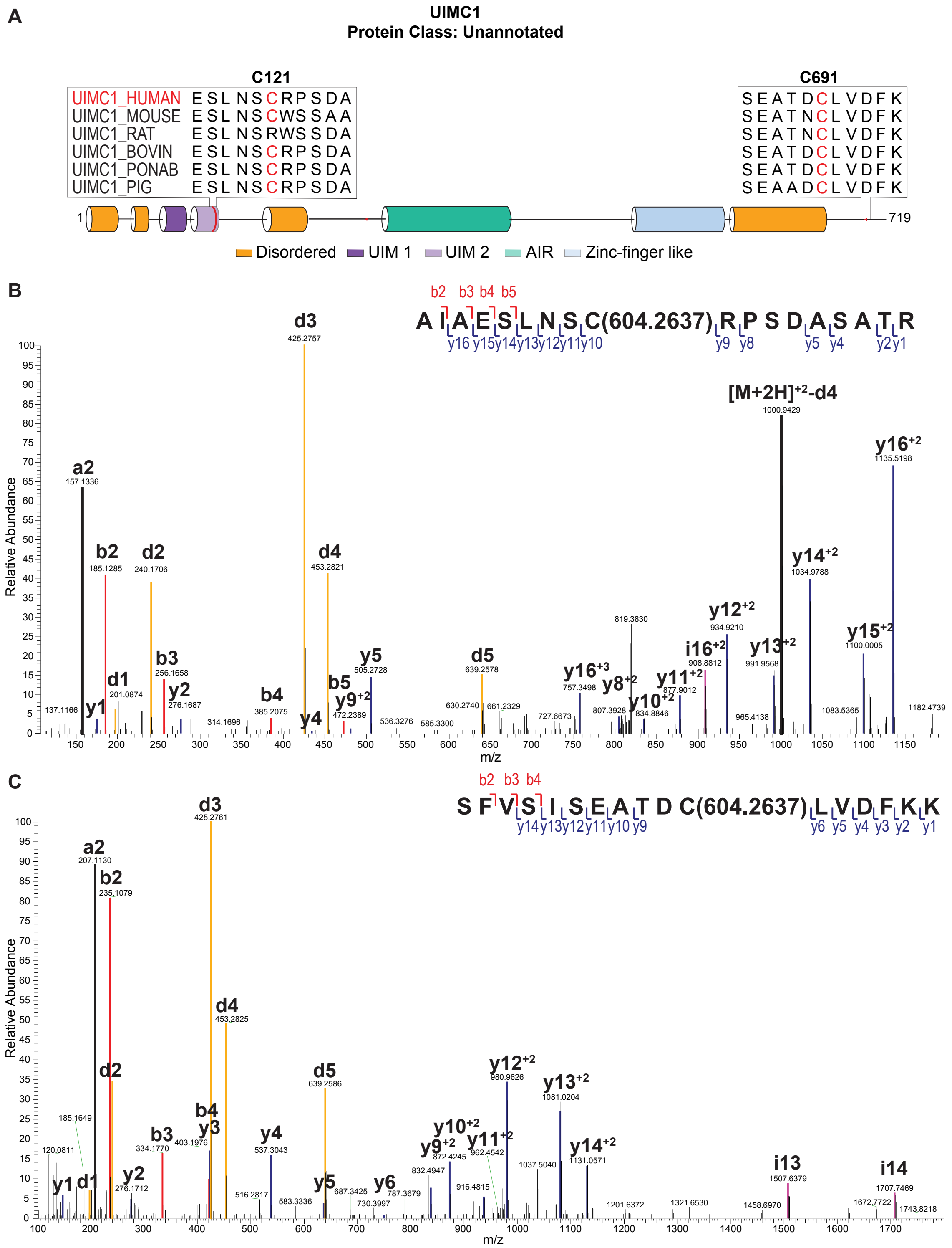

Supplementary Figure 19. **Mass Spectrometry analysis of the RS-Cys sites on UIMC1**. (A) UIMC1 domains and sequence alignments showing conservation of RS-Cys sites. (B) Modified sequence and MS2 fragment ion annotation of AIAESLNSC*RPSDASATR peptide from UIMC1 C121. (C) Modified sequence and MS2 fragment ion annotation of SFVSISEATDC*LVDFKK peptide from UIMC1 C691. See Supplementary Figure 15 for details on the annotation and interpretation of MS2 data.

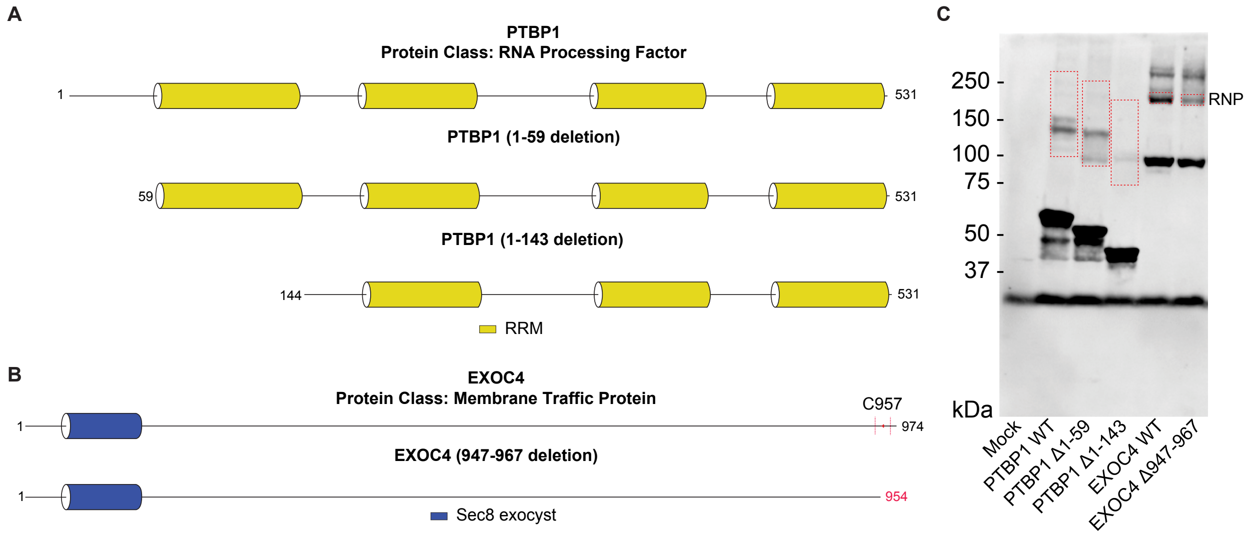

Supplementary Figure 20. **Analysis of RBP binding capacity using deletion mutants.** Diagram of PTBP1 (A) and EXOC4 (B) wild-type (WT) protein and domains/regions deleted in corresponding mutants. Red lines for EXOC4 represent a 20 amino acid deletion surrounding the RS-Cys C957 site. (C) Representative western blot comparing RNP formation of wild-type (WT) and corresponding RBP mutants in HEK293T cells used for quantitation. Data shown are representative of n=6 independent experiments.

2. CHEMICAL SYNTHESIS

General Information

Reagent grade chemicals were used without further purification. *N*,*N*-Dimethylformamide (DMF), ethyl acetate, chloroform and n-heptane were used without further purification (Fisher). Merck silica gel 60 F254 plates (0.25 mm) were used for analytical thin layer chromatography (TLC). Flash column chromatography was carried out using the indicated solvents on a Biotage Isolera One Automated flash chromatography purification system with UV detector (Uppsala, Sweden) using Teledyne ISCO columns. Compounds were visualized by UV-irradiation and iodine chamber. A Shimadzu 1100 Series spectrometer was used for Analytical HPLC chromatograms. Proton (^{1}H) and carbon (^{13}C) NMR spectra were recorded on a Varian Inova 500 (500 MHz) or 600 (600MHz) spectrometer in $CDCl_3$ or DMSO-d_6 with chemical shifts referenced to internal standards ($CDCl_3$: 7.26 ppm ^{1}H, 77.16 ppm ^{13}C; $(CD_3)_2SO$: 2.50 ppm ^{1}H, 39.52 ppm ^{13}C). Splitting patterns are indicated as follows: s, singlet; d, doublet; t, triplet; q, quartet; m, multiplet; br, broad singlet for 1H-NMR data. NMR chemical shifts (δ) are reported in ppm. Coupling constants (*J*) are reported in Hz. NMR studies were performed once per compound stock following standard synthetic protocols. An Agilent 6545B LC/Q-TOF (Agilent Technologies, Santa Clara, CA, USA) was used for high resolution mass spectral (HRMS) analysis. Structural analysis by crystallography was performed as previously described[5].

Chemical Suppliers

The chemicals listed below were purchased commercially and reported as ≥95% purity.

Fisher Scientific: *N,N*-Diisopropylethylamine, Acetic acid (optima LC/MS grade), Water (HPLC grade grade), and Acetonitrile (optima LC/MS grade)

Combi-Blocks: *p*-Cresol, *n*-Butylamine

Acros: 1,1,3,3-Tetramethylguanidine, 99% (TMG), Butyric acid, Propargyl bromide (80 wt% in toluene), Propanamide

Alfa Aesar: Caffeine

Oakwood Chemical: 1-Butanethiol

General procedure for the preparation of 2-iodo-*N*-(prop-2-yn-1-yl)acetamide (iodoacetamide alkyne)

Iodoacetamide alkyne was synthesized and characterized according to previously published literature[10].

General Protocol for synthesis of purine compounds

The purine base (21.7 mmol 1.0 eq), dimethylformamide (DMF, 100 mL), potassium carbonate (K_2CO_3, 21.7 mmol, 1.0 eq) and propargyl bromide (80 wt% in toluene, 21.7 mmol, 1.0 eq) were mixed in a round bottom flask. The reaction was stirred under nitrogen at room temperature for 12 hrs. The reaction was treated with water (400 mL) and extracted with ethyl acetate (3 x 100 mL). The combined organic layer was dried over sodium sulfate and concentrated to a tan solid. This solid was dissolved in chloroform (100 mL). The solution was concentrated and heated to reflux to dissolve all the solids. Upon cooling a white crystalline solid formed which was isolated by filtration. The solid was rinsed with fresh chloroform (20 mL) and heptane (20 mL) to give the N-9 substituted product after air drying. The filtrate contained a mixture of the N-7 and N-9 products. These were separated using the Biotage flash chromatography system (5% acetone to 20% acetone/chloroform) to afford respective products.

2,6-Dichloro-7-(prop-2-yn-1-yl)-7*H*-purine (AHL-Pu-1)

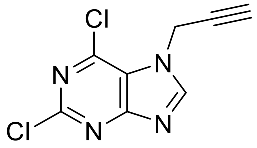

Yield: 9%. **^{1}H NMR** (600 MHz, $CDCl_3$) δ 8.48 (s, 1H), 5.27 (d, *J* = 2.6 Hz, 2H), 2.70 (t, *J* = 2.6 Hz, 1H). **^{13}C NMR** (151 MHz, DMSO-d6) δ 163.24, 151.84, 151.30, 143.40, 121.61, 78.08, 77.55, 36.62. ESI-TOF (HRMS) m/z $[M+H]^+$ calculated for $C_8H_5Cl_2N_4^+$ 226.9891, found 226.9885.

2,6-Dichloro-9-(prop-2-yn-1-yl)-9*H*-purine (AHL-Pu-2)

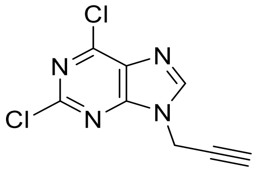

Yield: 45%. **^{1}H NMR** (600 MHz, $CDCl_3$) δ 8.33 (s, 1H), 5.04 (d, *J* = 2.6 Hz, 2H), 2.61 (t, *J* = 2.6 Hz, 1H). **^{13}C NMR** (151 MHz, DMSO-d_6) δ 152.91, 151.21, 149.89, 147.71, 130.44, 77.07, 76.92, 33.52. ESI-TOF (HRMS) m/z $[M+H]^+$ calculated for $C_8H_5Cl_2N_4^+$ 226.9891, found 226.9887.

General procedure for the preparation of 1-alkylthiol adducts

Dichloropurine compound (831 mg, 3.66 mmol), DMF (10 mL), potassium carbonate (powdered, 556 mg, 4.03 mmol) and *n*-butanethiol (373 mg, 4.14 mmol) were placed in a 50 mL round bottom flask. The reaction was stirred under nitrogen at ambient temperature for 16 hrs. The reaction was partitioned between ethyl acetate and water (25 mL/40 mL). The layers were separated, and the aqueous layer was extracted with ethyl acetate (2 x 25 mL). The combined organic layer was washed with water (25 mL) and brine (25 mL). It was then dried over magnesium sulfate and

concentrated to give crude product that was purified on the Biotage flash chromatography system (20% ethyl acetate to 80% hexanes) to give 650 mg of product as an off-white solid.

6-(Butylthio)-2-chloro-7-(prop-2-yn-1-yl)-7H-purine (Pa-1)

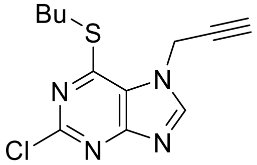

Yield: 63%. **^{1}H NMR** (600 MHz, dmso) δ 8.68 (s, 1H), 5.32 (d, *J* = 2.5 Hz, 2H), 3.67 (t, *J* = 2.5 Hz, 1H), 3.39 – 3.36 (m, 2H), 1.74 – 1.68 (m, 2H), 1.44 (dq, *J* = 14.7, 7.4 Hz, 2H), 0.92 (t, *J* = 7.4 Hz, 3H). **^{13}C NMR** (151 MHz, dmso) δ 159.97, 155.92, 152.44, 149.41, 121.69, 78.26, 77.88, 36.97, 30.57, 29.11, 21.24, 13.43. ESI-TOF (HRMS): m/z [M+H]+ calculated for $C_{12}H_{14}ClN_4S^+$ 281.0622, found 281.0621.

Purity of CEP probes

CEP probes were prepared in ACN (10 mM final). The compound stock (50 µL) was then mixed with 10 µL of ACN. This sample mixture was injected (1 µL) and analyzed by reverse-phase HPLC on a Shimadzu 1100 Series spectrometer with UV detection at 254 nm. Chromatographic separation was performed using a Phenomenex Kinetex C18 column (2.6 µM, 50 x 4.6 mm). Mobile phases A was composed of H_2O + 0.1% HOAc, while B was composed of ACN + 0.1% HOAc, respectively. Samples were analyzed using the following analytical conditions: 0–0.5 min, 15% B; 0.5–6.5 min 85% B; 6.5–7 min 100% B; 7–8.5 min 100% B; 8.5–9 min 15% B; 9–9.8 min 15% B using a flow rate of 0.8 mL min^{-1}.

HPLC solution reactivity assay

The following reagents were prepared and stored on ice prior to use. 0.1 molar (M) solution of caffeine in acetonitrile, 1.0 M solution of nucleophile, tetramethylguanidine (TMG), 1 M acetic acid (HOAc) in acetonitrile (ACN) and 10 mM solution of CEP probe in ACN. The following nucleophiles that mimic amino acid side chain groups were used: butanethiol (Cys mimetic), *n*-butylamine (Lys mimetic), *p*-cresol (Tyr mimetic), propionamide (Asn/Gln mimetic); butyric acid (Asp/Glu mimetic). The CEP solution (500 μL) was transferred to a dram vial on ice. TMG (5.5 μL) and the respective nucleophile (5.5 μL) were added and the solutions stirred on ice for 6 hr. Aliquots (50 μL) were removed at the indicated time points and quenched with 10 μL of a 1:1 mixture of caffeine and HOAc to monitor probe reactivity. Reaction progress was evaluated by monitoring consumption of starting material (CEP) normalized to the caffeine standard. CEP consumption was calculated using the area under the curve (AUC) for the CEP peak at time (t) = experimental / t = 0. All CEP peak AUCs used for calculations were normalized to caffeine standard AUCs at respective time points to account for run-to-run variations by HPLC. The amount of CEP consumed (% starting material) was plotted as a function of time. LC parameters were followed as described in the purity of CEP probes section.

3. APPENDIX
3.1 HPLC analysis of compound purity and stability

HPLC Method A: chromatographic separation was performed using a Phenomenex Kinetex C18 column (2.6 µm, 50 x 4.6 mm). Mobile phases A and B were composed of H_2O + 0.1% HOAc and ACN + 0.1% HOAc, respectively. Samples were analyzed using the following analytical conditions: using a flow rate of 0.8 mL min−1, the gradient was as follows: 0–0.5 min, 15% B; 0.5– 6.5 min 85% B; 6.5–7 min 100% B; 7–8.5 min 100% B; 8.5–9 min 15% B; 9–9.8 min 15% B.

A

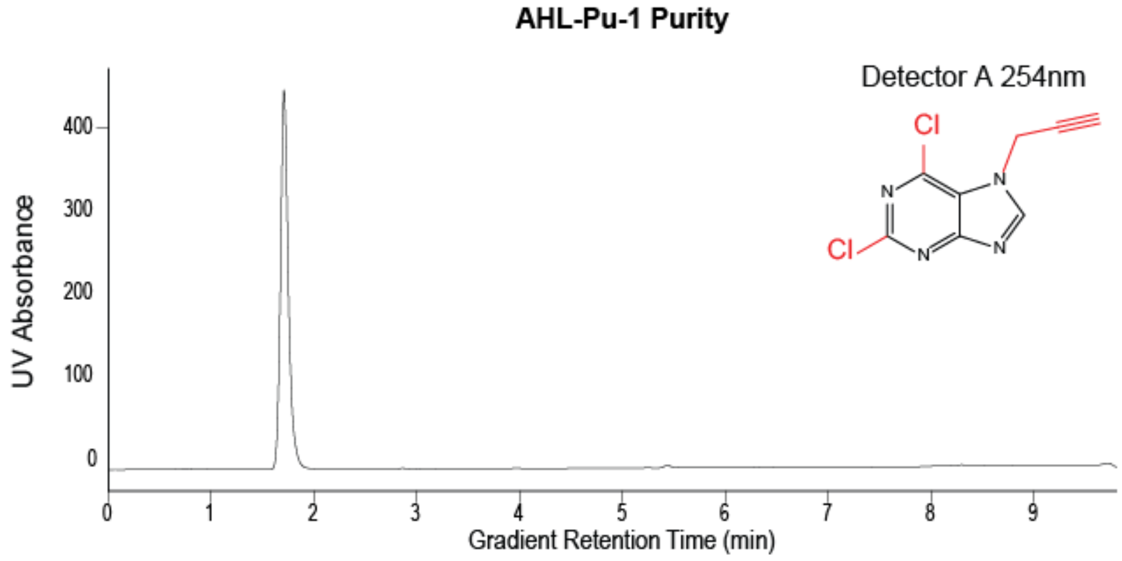

B

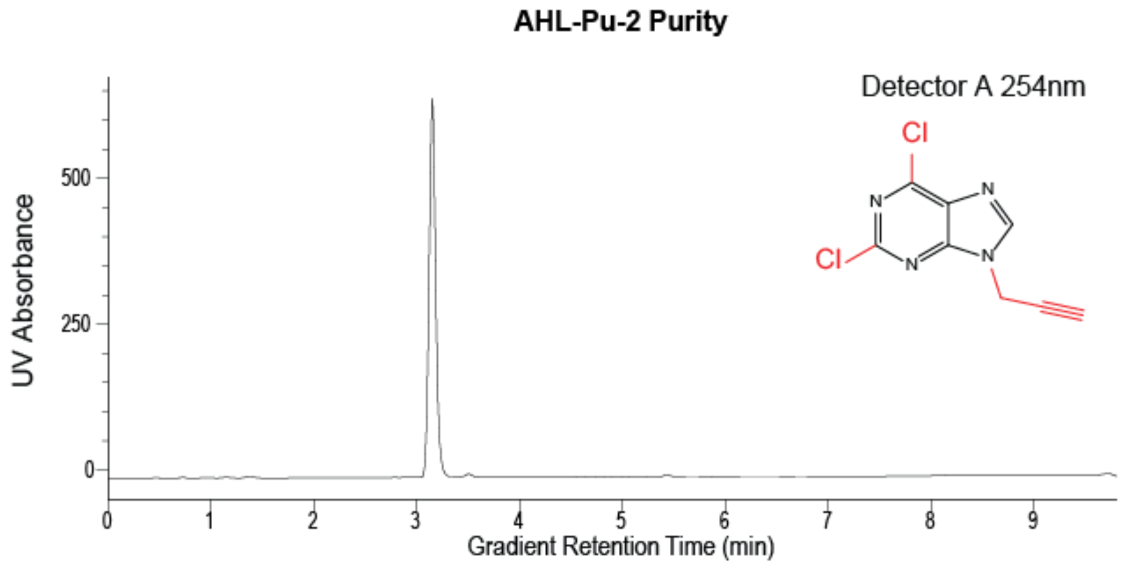

The purity of AHL-Pu-1 (A) and AHL-Pu-2 (B) was determined to be ≥95% by using HPLC method A.

AHL-Pu-1 Butanethiol 6-Position Substitution Purity

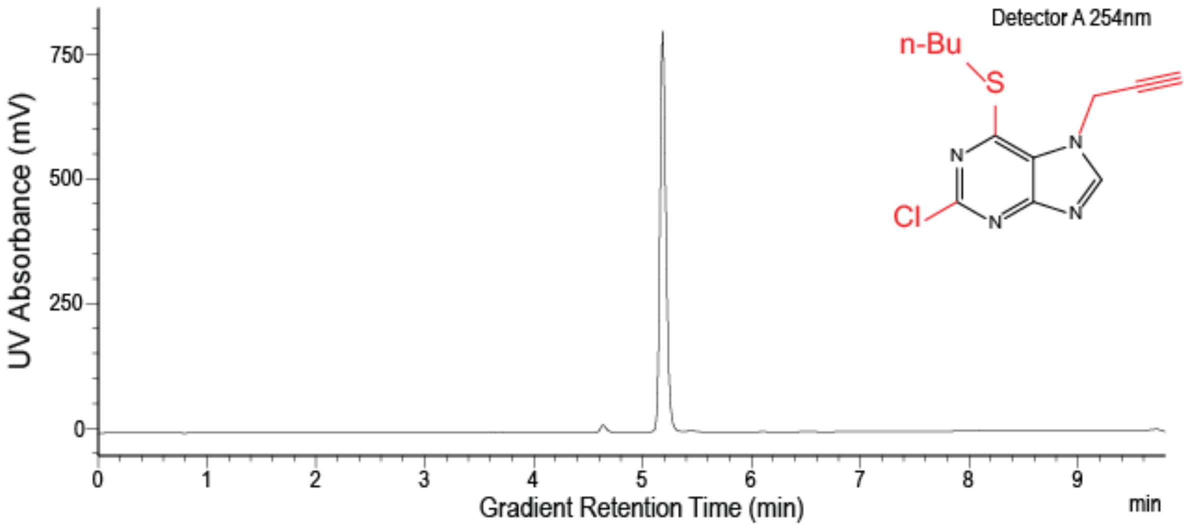

The purity of Pa-1 was determined to be ≥95% by using HPLC method A.

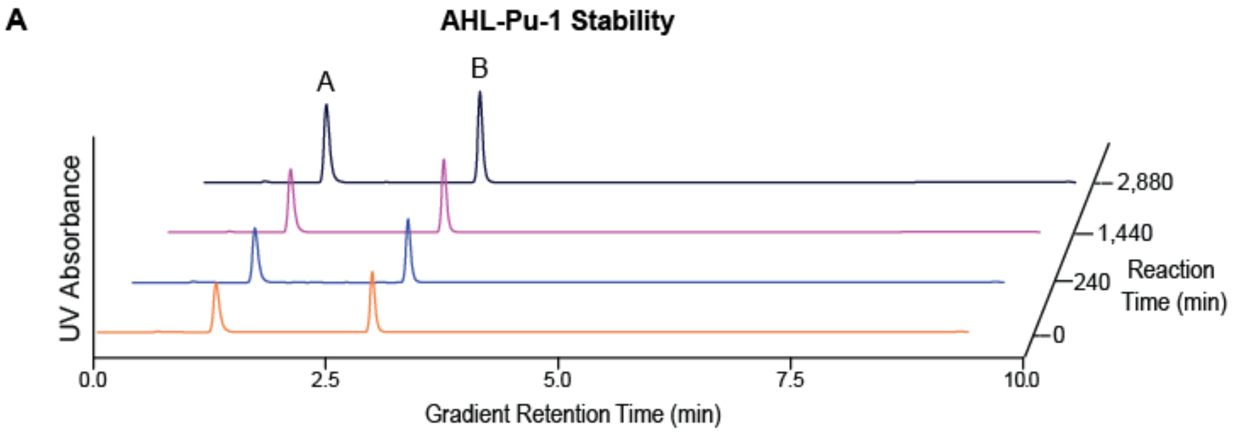

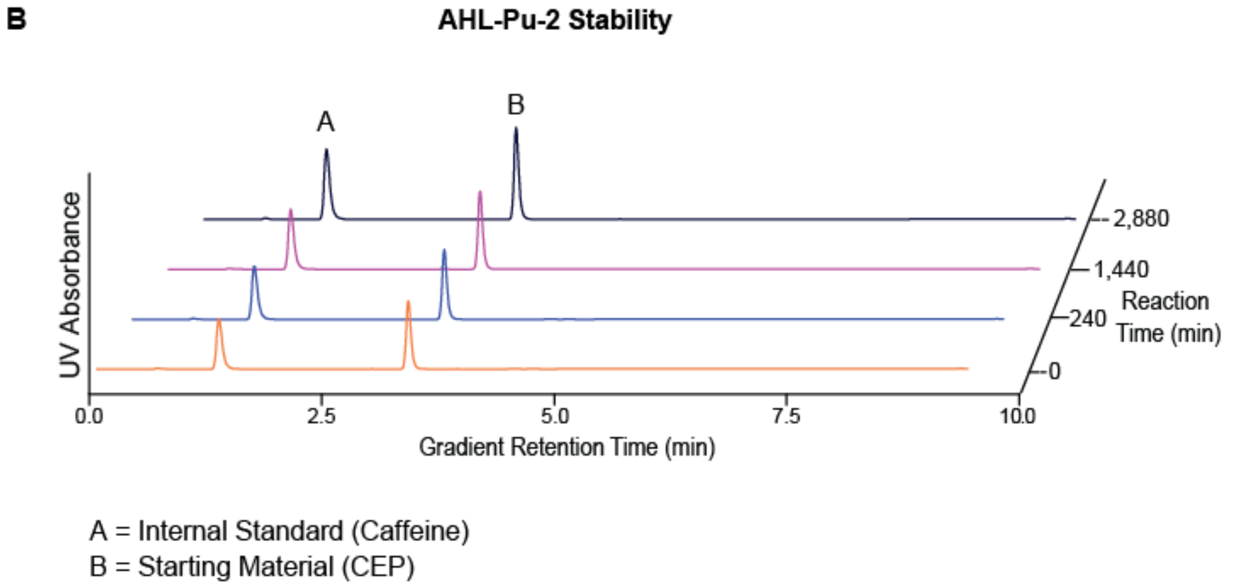

(A) AHL-Pu-1 PBS stability chromatograms. Compound integrity after 48 hrs was determined to be >99% by HPLC method A. (B) AHL-Pu-2 PBS stability chromatograms. Compound integrity after 48 hrs was determined to be >92% by HPLC method A.

3.2 HPLC analysis of compound reactivity

HPLC Method B: Probes were dissolved in 500 μL DMF-ACN solution and stirred on ice with TMG and the amino acid mimetics. At the indicated time point, a 50 μL aliquot was removed and quenched in a solution of acetic acid and caffeine. Solutions were analyzed by HPLC and consumption of CEP probe was quantified as described in the Methods section. The HPLC gradient from Method A was used in these assays.

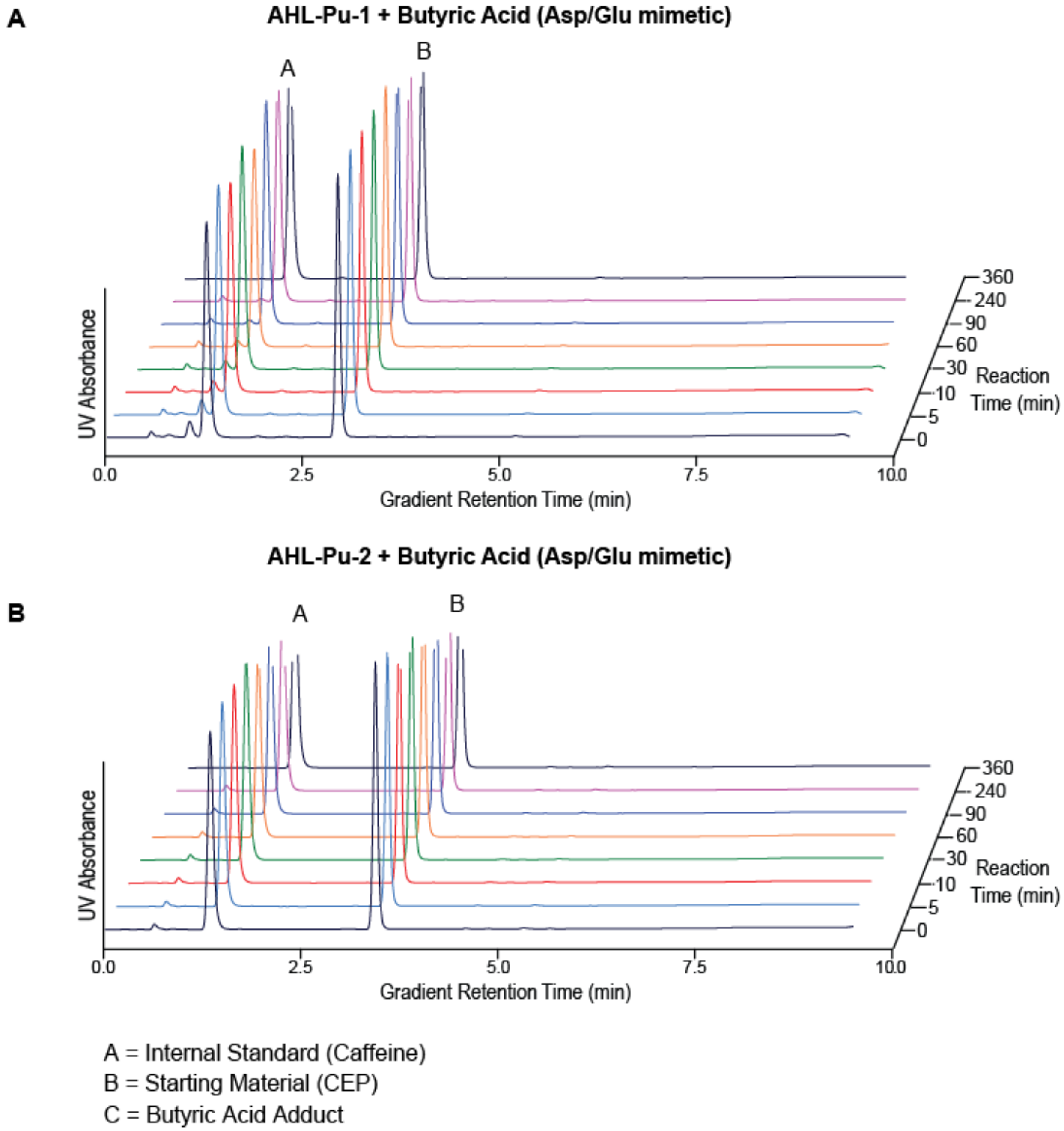

(A) HPLC chromatograms of AHL-Pu-1 in the presence of butyric acid (Asp/Glu mimetic). (B) HPLC chromatograms of AHL-Pu-2 in the presence of butyric acid (Asp/Glu mimetic). Consumption of the probe was analyzed as described in HPLC Method B and quantified as described in the Methods section.

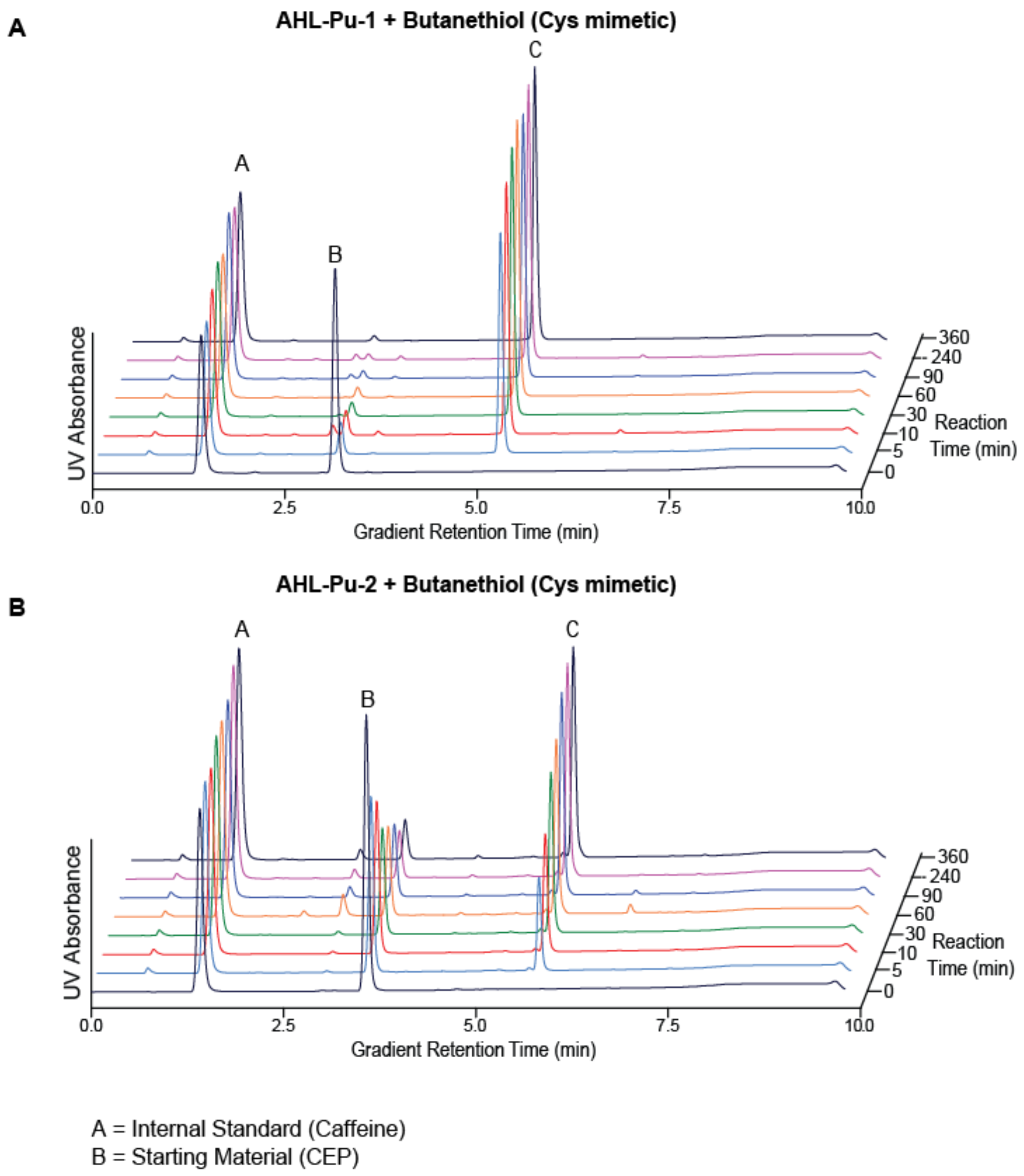

(A) HPLC chromatograms of AHL-Pu-1 in the presence of butanethiol (Cys mimetic). (B) HPLC chromatograms of AHL-Pu-2 in the presence of butanethiol (Cys mimetic). Consumption of the probe was analyzed as described in HPLC Method B and quantified as described in the Methods section.

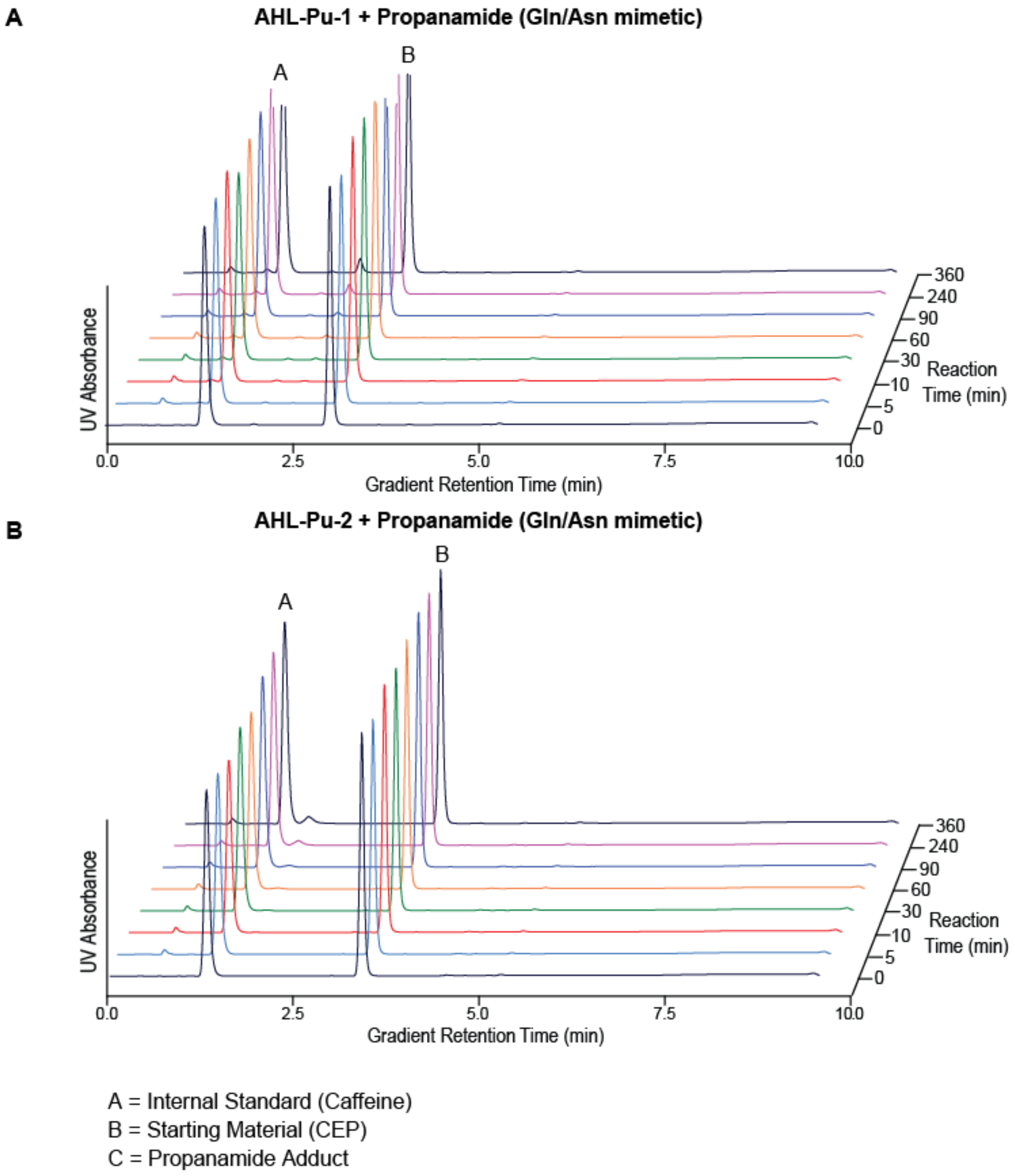

(A) HPLC chromatograms of AHL-Pu-1 in the presence of propanamide (Gln/Asn mimetic). (B) HPLC chromatograms of AHL-Pu-2 in the presence of propanamide (Gln/Asn mimetic). Consumption of the probe was analyzed as described in HPLC Method B and quantified as described in the Methods section.

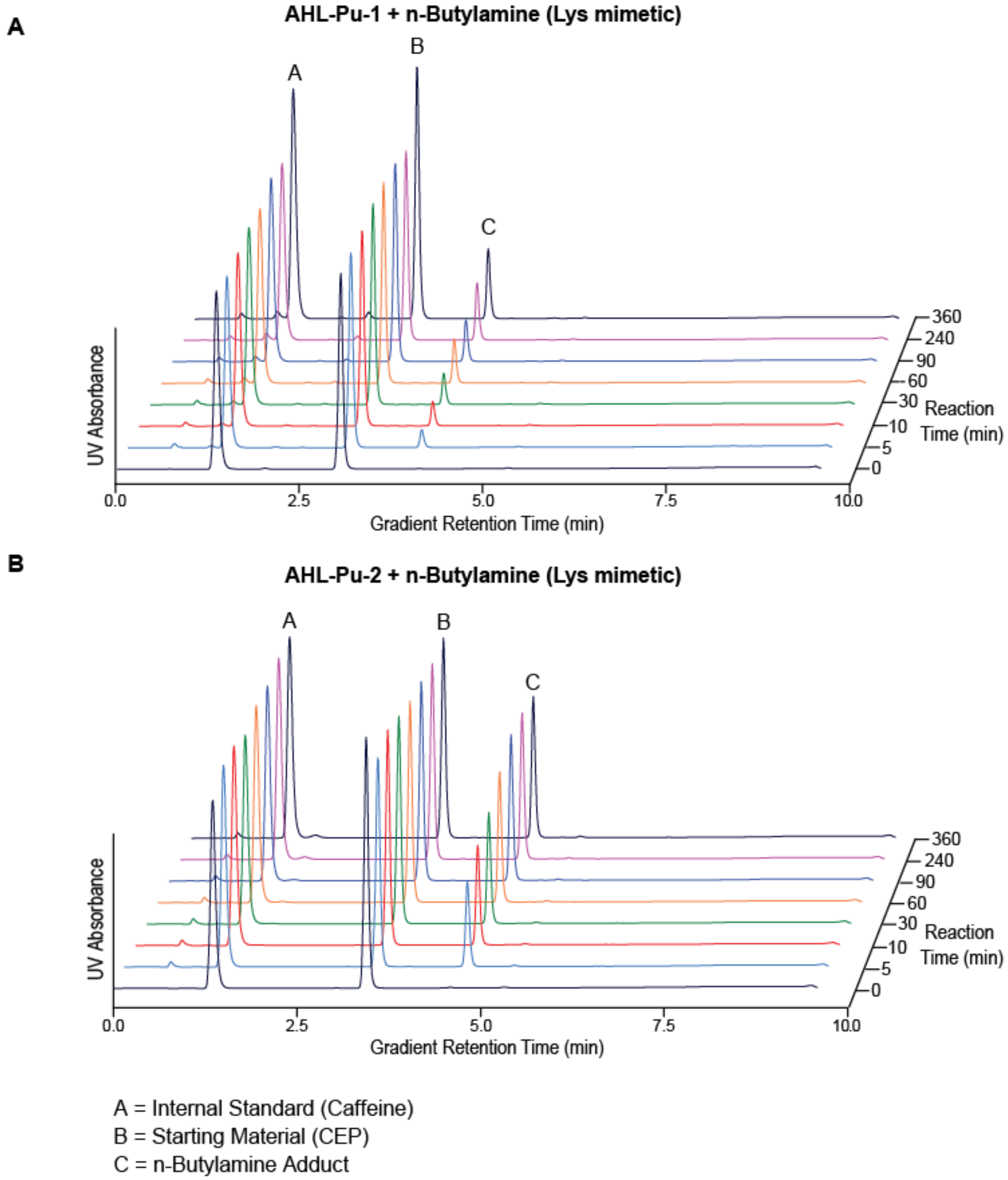

(A) HPLC chromatograms of AHL-Pu-1 in the presence of n-butylamine (Lys mimetic). (B) HPLC chromatograms of AHL-Pu-2 in the presence of n-butylamine (Lys mimetic). Consumption of the probe was analyzed as described in HPLC Method B and quantified as described in the Methods section.

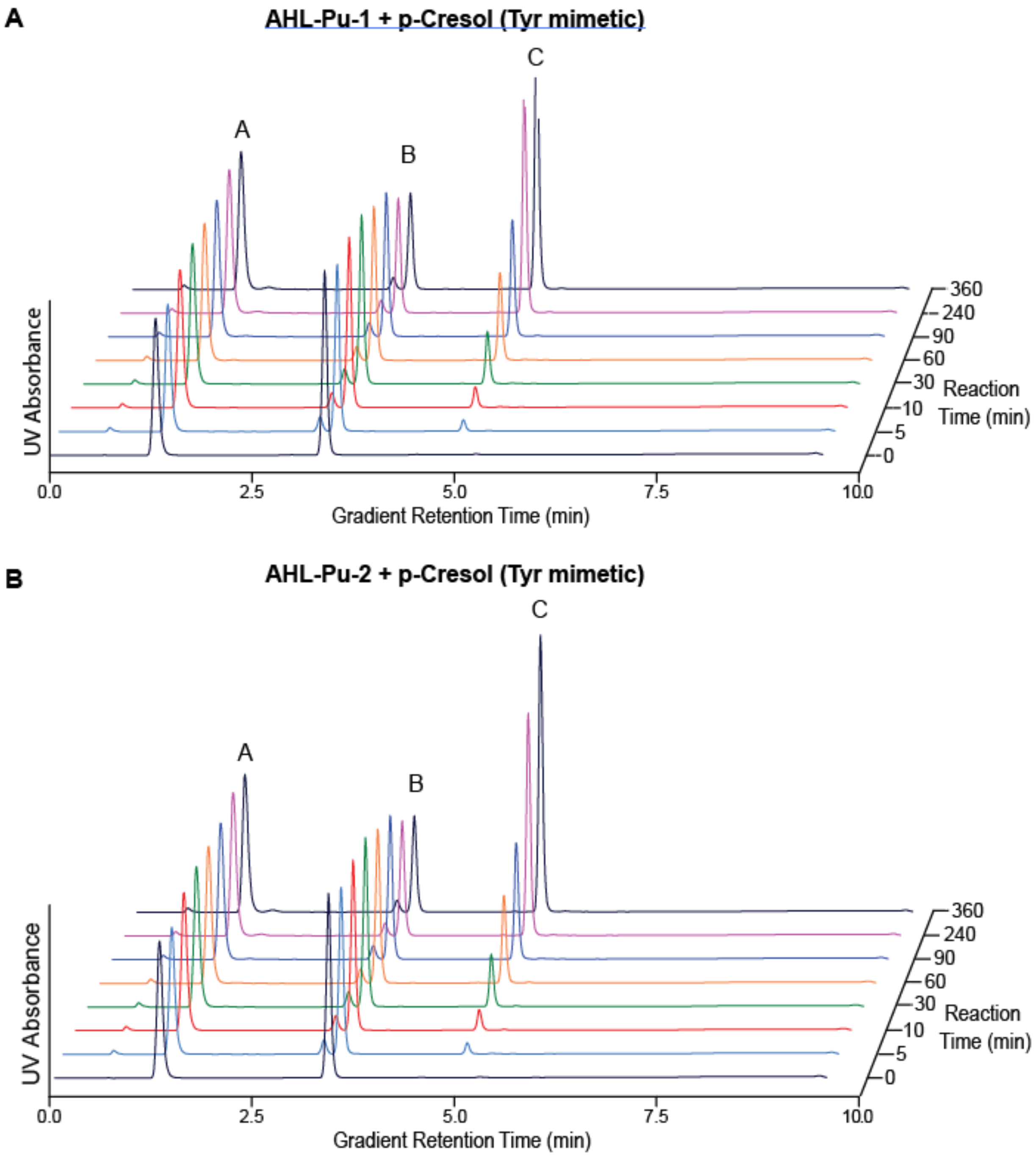

A = Internal Standard (Caffeine)
B = Starting Material (CEP)
C = p-Cresol Adduct

(A) HPLC chromatograms of AHL-Pu-1 in the presence of p-cresol (Tyr mimetic). (B) HPLC chromatograms of AHL-Pu-2 in the presence of n-butylamine p-cresol (Tyr mimetic). Consumption of the probe was analyzed as described in HPLC Method B and quantified as described in the Methods section.

3.3 NMR Spectra

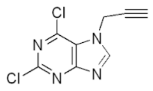

AHL-Pu-1

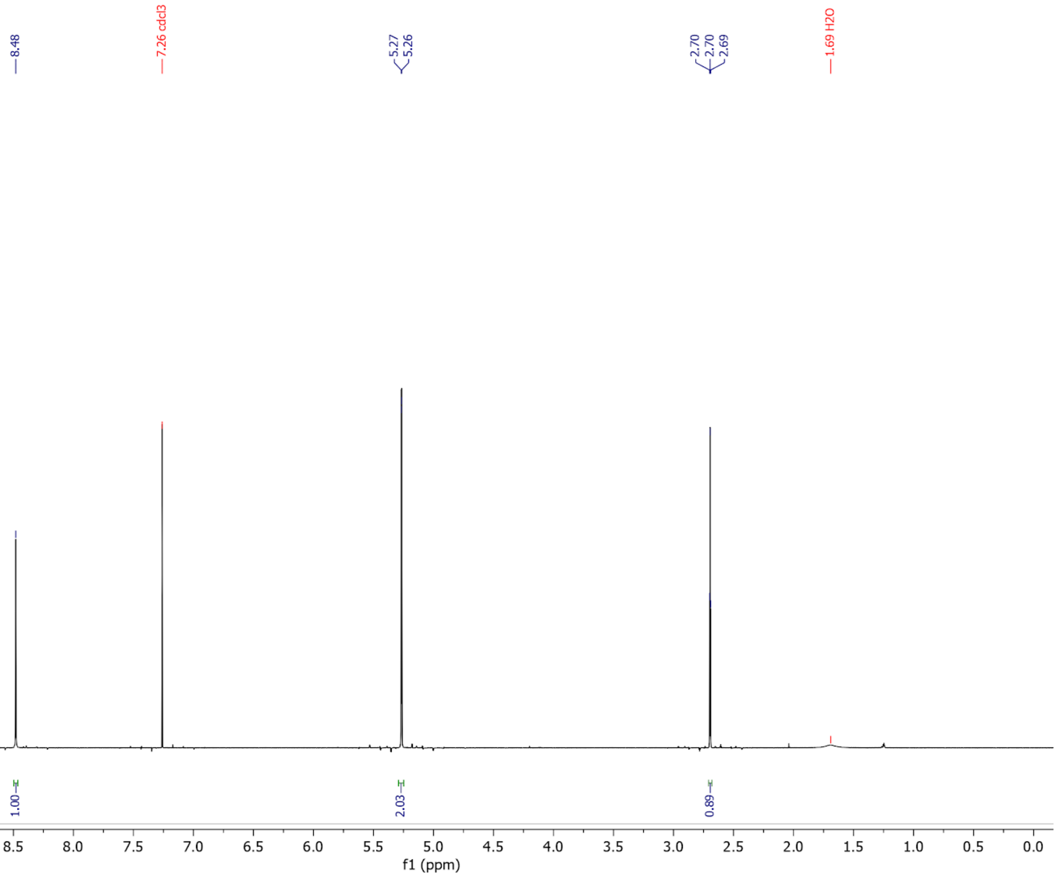

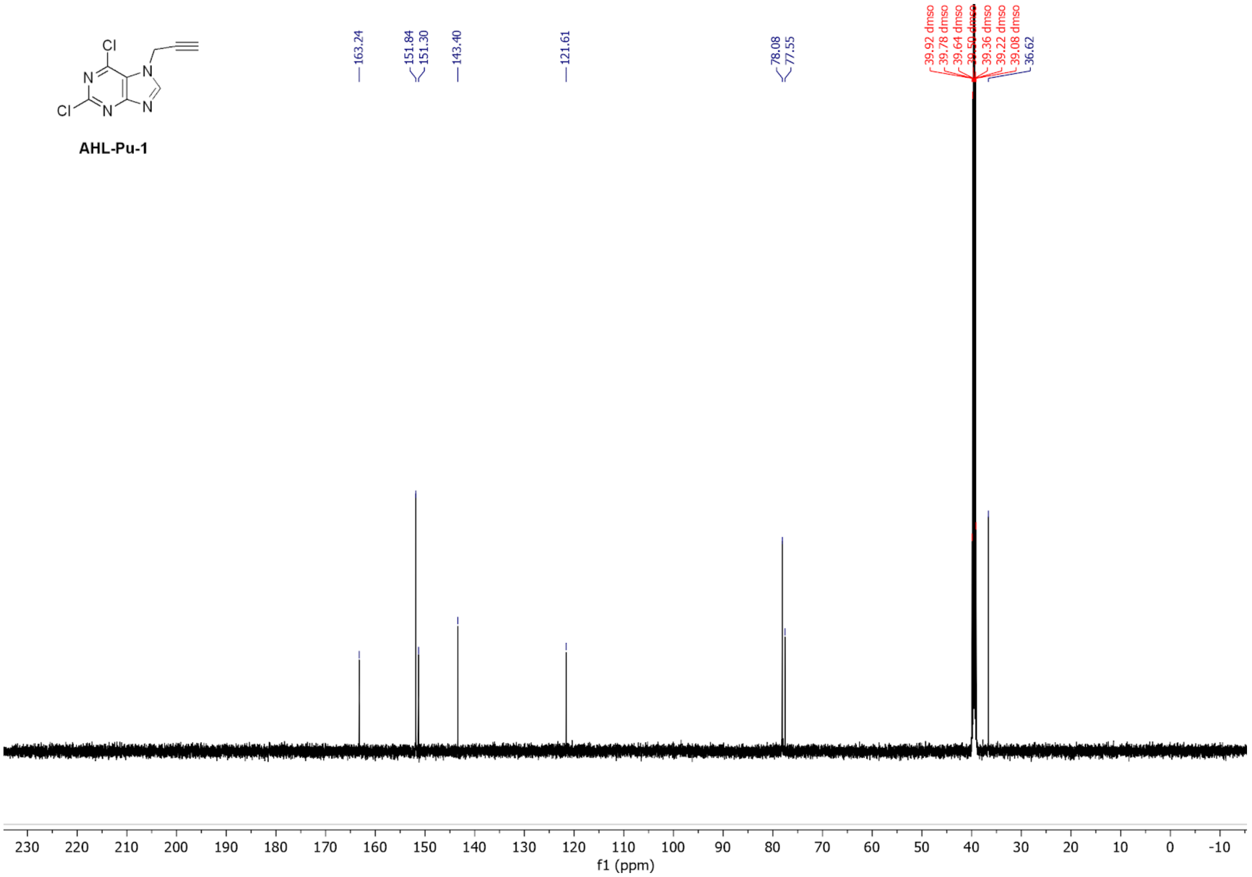
AHL-Pu-1
163.24
151.84
151.30
143.40
121.61
78.08
77.55
39.92 dmso
39.78 dmso
39.64 dmso
39.50 dmso
39.36 dmso
39.22 dmso
39.08 dmso
36.62
f1 (ppm)

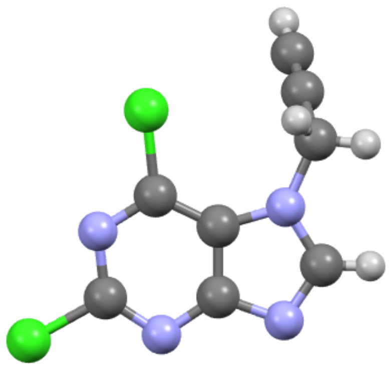

Structure of **AHL-Pu-1** derived from X-ray crystallographic analysis. AHL-Pu-1 matches a previously reported crystal structure[11].

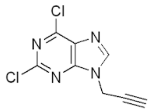

AHL-Pu-2

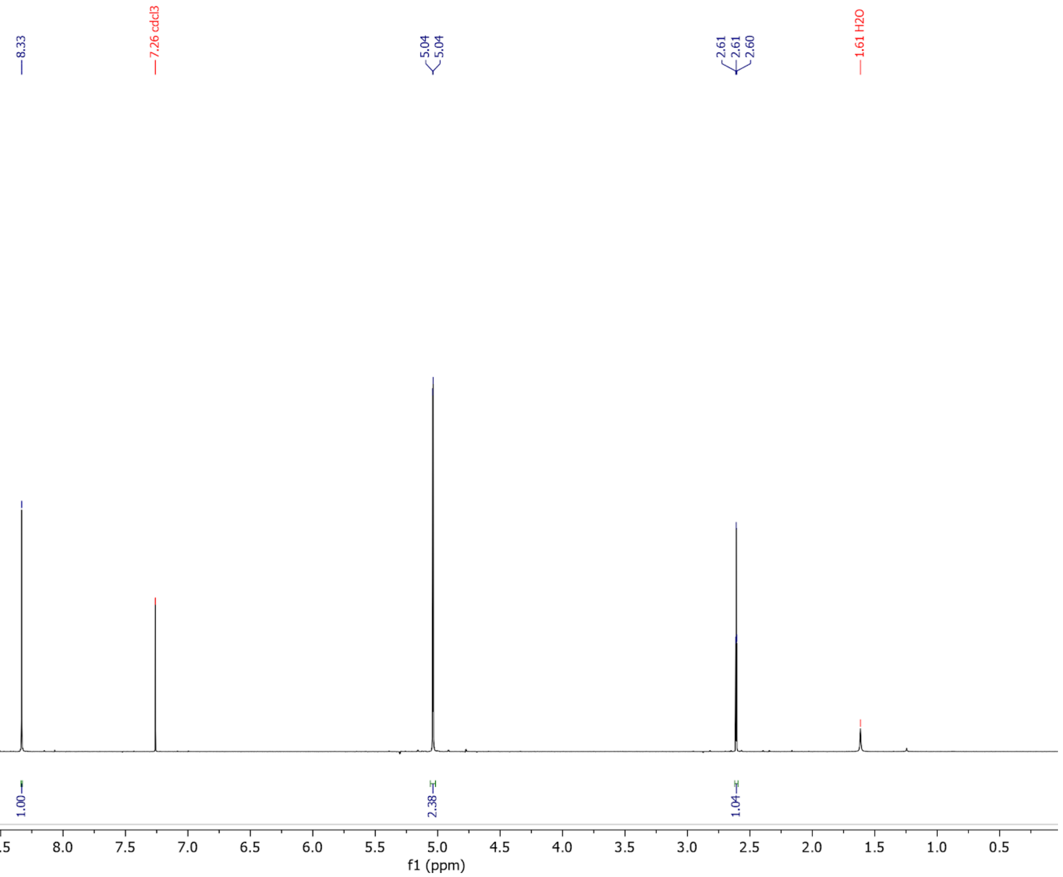

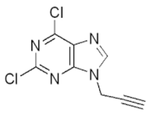
Cl
N
N
Cl
N
N

AHL-Pu-2

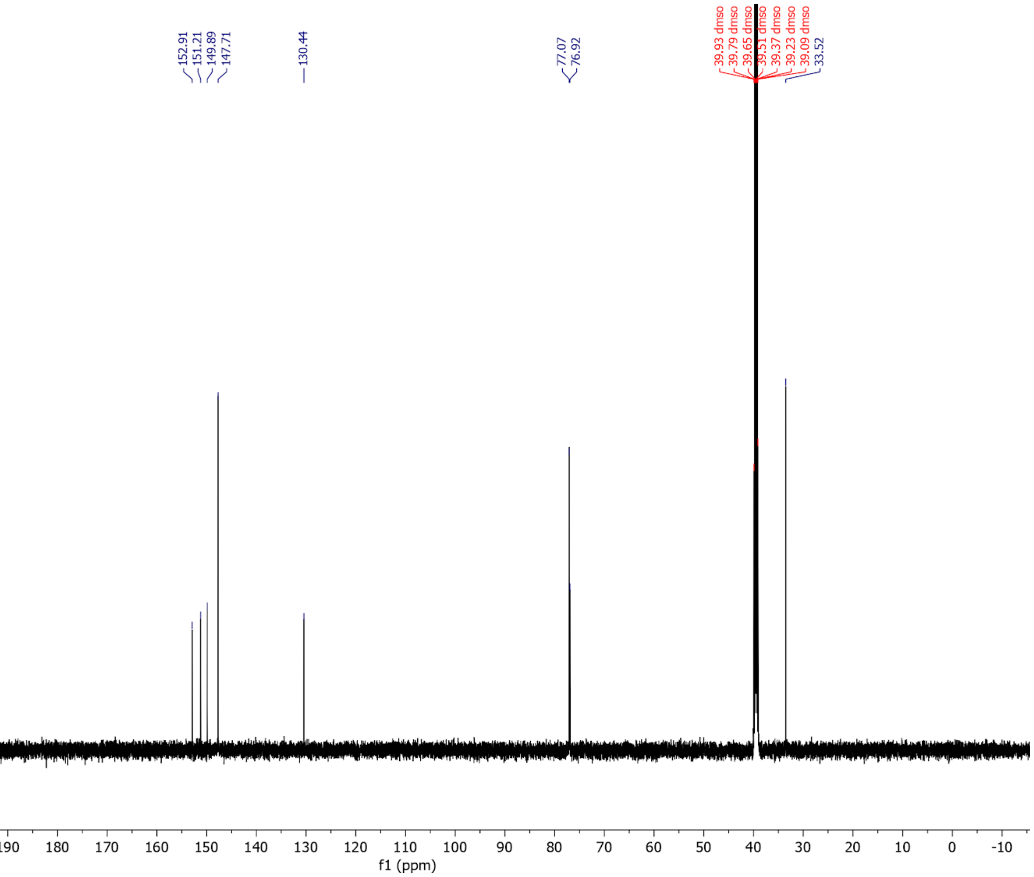
152.91
151.21
149.89
147.71
130.44
77.07
76.92
39.93 dmso
39.79 dmso
39.65 dmso
39.51 dmso
39.37 dmso
39.23 dmso
39.09 dmso
33.52
230 220 210 200 190 180 170 160 150 140 130 120 110 100 90 80 70 60 50 40 30 20 10 0 -10
f1 (ppm)

*Crystallography was performed on **AHL-Pu-2** and the unit cell determination showed an exact match to a previously published compound that is consistent with the expected product[12] (**CCDC 638951**).

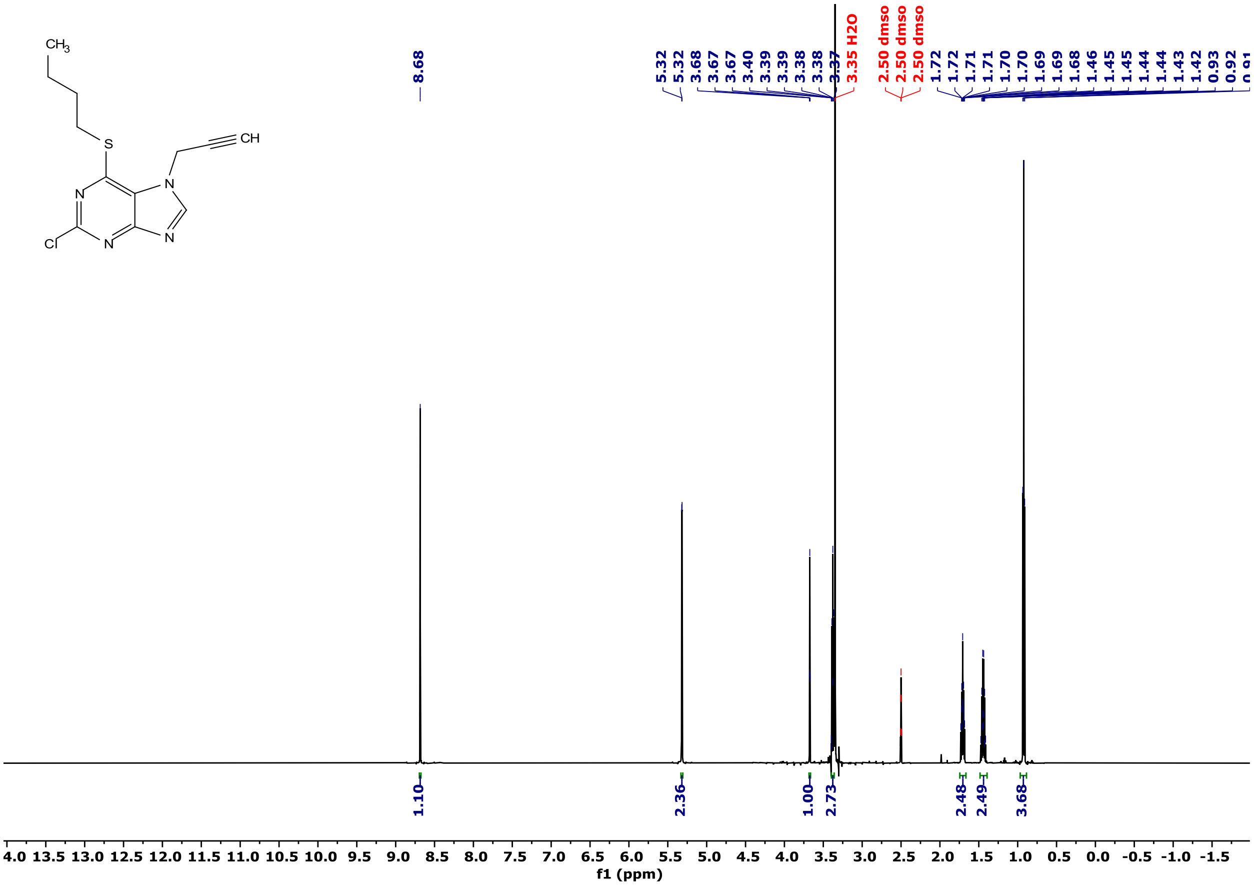

CH3
S
CH
N
N
Cl
N
N
8.68
5.32
5.32
3.68
3.67
3.67
3.40
3.39
3.39
3.38
3.38
3.37
3.35 H2O
2.50 dmso
2.50 dmso
2.50 dmso
1.72
1.72
1.71
1.71
1.70
1.70
1.69
1.69
1.68
1.46
1.45
1.45
1.44
1.44
1.43
1.42
0.93
0.92
1.10
2.36
1.00
2.73
2.48
2.49
3.68
4.0 13.5 13.0 12.5 12.0 11.5 11.0 10.5 10.0 9.5 9.0 8.5 8.0 7.5 7.0 6.5 6.0 5.5 5.0 4.5 4.0 3.5 3.0 2.5 2.0 1.5 1.0 0.5 0.0 -0.5 -1.0 -1.5
f1 (ppm)

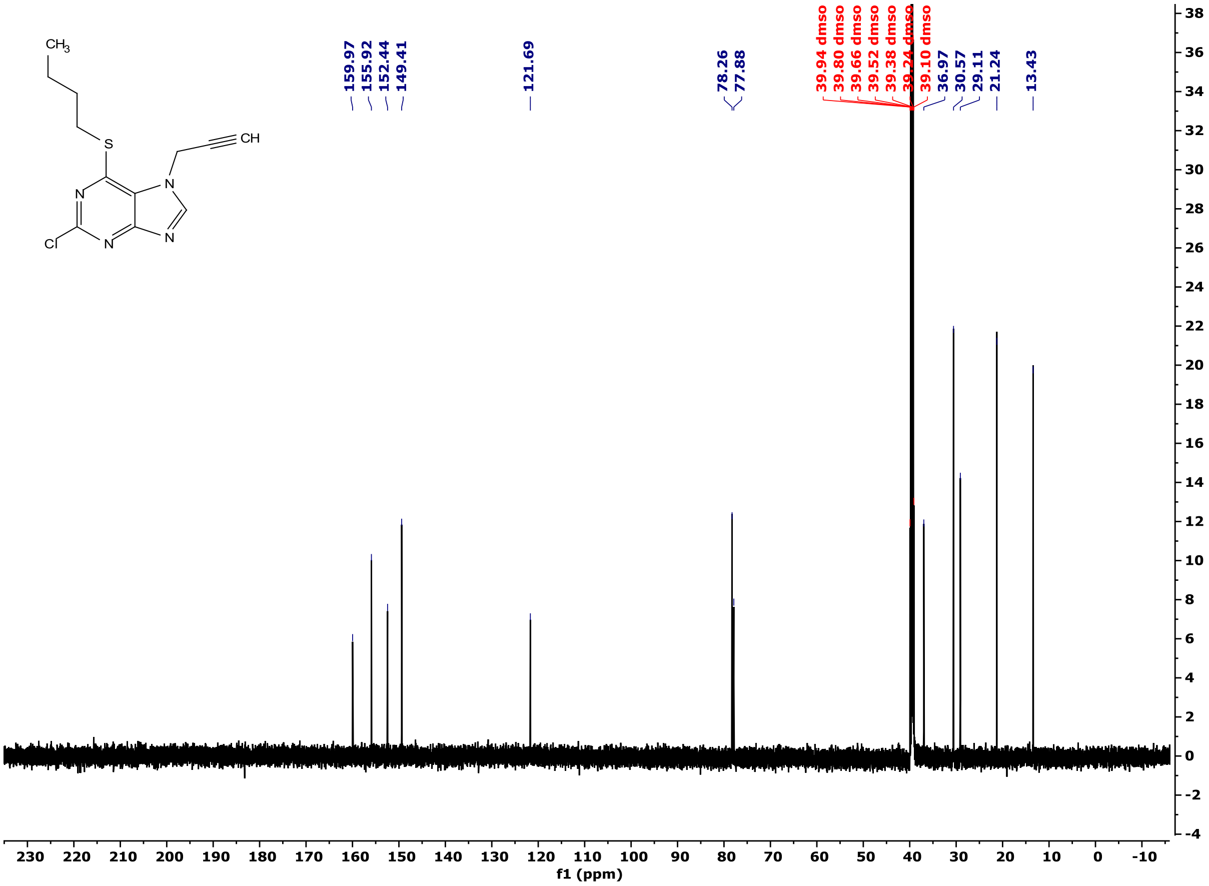
CH3
S
CH
N
N
N
N
Cl
159.97
155.92
152.44
149.41
121.69
78.26
77.88
39.94 dmso
39.80 dmso
39.66 dmso
39.52 dmso
39.38 dmso
39.24 dmso
39.10 dmso
36.97
30.57
29.11
21.24
13.43
f1 (ppm)

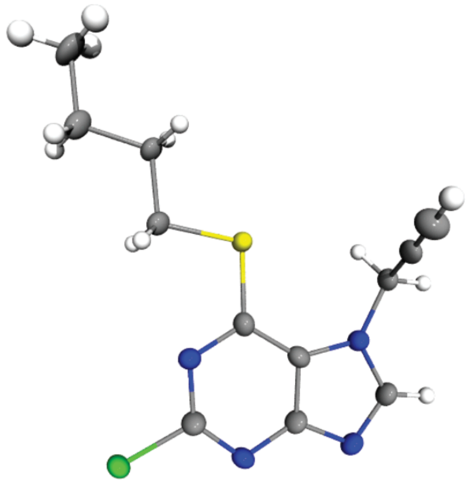

Structure of **Pa-1** derived from X-ray crystallographic analysis.

4. References

1. Ashburner, M. et al. Gene ontology: tool for the unification of biology. The Gene Ontology Consortium. *Nat Genet* **25**, 25-29 (2000).
2. Gene Ontology, C. The Gene Ontology resource: enriching a GOld mine. *Nucleic Acids Res* **49**, D325-D334 (2021).
3. Huang, R., Han, M., Meng, L. & Chen, X. Transcriptome-wide discovery of coding and noncoding RNA-binding proteins. *Proc Natl Acad Sci U S A* **115**, E3879-E3887 (2018).
4. Biasini, A. & Marques, A.C. A Protocol for Transcriptome-Wide Inference of RNA Metabolic Rates in Mouse Embryonic Stem Cells. *Front Cell Dev Biol* **8**, 97 (2020).
5. Brulet, J.W., Borne, A.L., Yuan, K., Libby, A.H. & Hsu, K.L. Liganding Functional Tyrosine Sites on Proteins Using Sulfur-Triazole Exchange Chemistry. *J Am Chem Soc* **142**, 8270-8280 (2020).
6. Baltz, A.G. et al. The mRNA-bound proteome and its global occupancy profile on protein-coding transcripts. *Mol Cell* **46**, 674-690 (2012).
7. Castello, A. et al. Comprehensive Identification of RNA-Binding Domains in Human Cells. *Mol Cell* **63**, 696-710 (2016).
8. Castello, A. et al. Insights into RNA biology from an atlas of mammalian mRNA-binding proteins. *Cell* **149**, 1393-1406 (2012).
9. Conrad, T. et al. Serial interactome capture of the human cell nucleus. *Nat Commun* **7**, 11212 (2016).
10. Gless, B.H. & Olsen, C.A. Direct Peptide Cyclization and One-Pot Modification Using the MeDbz Linker. *J Org Chem* **83**, 10525-10534 (2018).
11. Khazir, J., Mir, B.A., Chashoo, G., Pilcher, L. & Riley, D. Synthesis and anticancer activity of N-9- and N-7- substituted 1,2,3 triazole analogues of 2,6-di-substituted purine. *Med Chem Res* **29**, 33-45 (2020).
12. Lu, W., Sengupta, S., Petersen, J.L., Akhmedov, N.G. & Shi, X. Mitsunobu coupling of nucleobases and alcohols: an efficient, practical synthesis for novel nonsugar carbon nucleosides. *J Org Chem* **72**, 5012-5015 (2007).
