# Supplementary material for: Chemoproteomic capture of RNA binding activity in living cells
Source: Nat Commun. 2023 Oct 7;14:6282. doi: 10.1038/s41467-023-41844-z (PMC10560261; doi:10.1038/s41467-023-41844-z)
Supplement: Supplementary file 3 — Description of Additional Supplementary Files [file 41467_2023_41844_MOESM3_ESM.pdf]

Description of Additional Supplementary Files

File Name: Supplementary Data 1
Description: Data used for chemoproteomic, domain enrichment, Gene Ontology and Panther classification analyses of AHL-Pu-1, AHL-Pu-2 and iodoacetamide alkyne.

File Name: Supplementary Data 2
Description: Databases used in RNA comparisons and SILAC datasets for chemoproteomic studies

File Name: Supplementary Data 3
Description: Domain and molecular function enrichment for chemoproteomic studies. Protein class enrichments were conducted on default settings: Fisher's exact test, Benjamini-Hochberg false discovery rate (FDR) < 0.05. GO Enrichment analysis utilized molecular function analysis on the following settings: binomial test, FDR correction

File Name: Supplementary Data 4
Description: Data obtained from PACCE studies.

File Name: Supplementary Data 5
Description: Domain, molecular function, and Panther classification analysis for PACCE datasets. Default settings (Fisher's exact test, Benjamini-Hochberg false discovery rate (FDR) < 0.05) were used for protein class enrichments. Molecular function analysis utilized the following settings: binomial test with a FDR correction.

File Name: Supplementary Data 6
Description: DDX proteins and their functions.

File Name: Supplementary Data 7
Description: Distance and domain information for PACCE datasets.
