# Supplementary material for: Chemoproteomic capture of RNA binding activity in living cells
Source: Nat Commun. 2023 Oct 7;14:6282. doi: 10.1038/s41467-023-41844-z (PMC10560261; doi:10.1038/s41467-023-41844-z)
Supplement: Supplementary file 11 — Reporting Summary [file 41467_2023_41844_MOESM11_ESM.pdf]

nature research

Corresponding author(s): Ku-Lung Hsu

Last updated by author(s): Sep 1, 2023

Reporting Summary

Nature Research wishes to improve the reproducibility of the work that we publish. This form provides structure for consistency and transparency in reporting. For further information on Nature Research policies, see our Editorial Policies and the Editorial Policy Checklist.

Statistics

For all statistical analyses, confirm that the following items are present in the figure legend, table legend, main text, or Methods section.

n/a	Confirmed	
☐	☒	The exact sample size (*n*) for each experimental group/condition, given as a discrete number and unit of measurement
☐	☒	A statement on whether measurements were taken from distinct samples or whether the same sample was measured repeatedly
☐	☒	The statistical test(s) used AND whether they are one- or two-sided<br>*Only common tests should be described solely by name; describe more complex techniques in the Methods section.*
☒	☐	A description of all covariates tested
☐	☒	A description of any assumptions or corrections, such as tests of normality and adjustment for multiple comparisons
☐	☒	A full description of the statistical parameters including central tendency (e.g. means) or other basic estimates (e.g. regression coefficient) AND variation (e.g. standard deviation) or associated estimates of uncertainty (e.g. confidence intervals)
☐	☒	For null hypothesis testing, the test statistic (e.g. *F*, *t*, *r*) with confidence intervals, effect sizes, degrees of freedom and *P* value noted<br>*Give P values as exact values whenever suitable.*
☒	☐	For Bayesian analysis, information on the choice of priors and Markov chain Monte Carlo settings
☒	☐	For hierarchical and complex designs, identification of the appropriate level for tests and full reporting of outcomes
☒	☐	Estimates of effect sizes (e.g. Cohen's *d*, Pearson's *r*), indicating how they were calculated

Our web collection on statistics for biologists contains articles on many of the points above.

Software and code

Policy information about availability of computer code

Data collection	Thermo Fisher Q--Exactive Series (v.2.8 SP1)
Data analysis	Byonic mass spectrometry search software (v.2.3.0 and 4.1.1), Skyline Targeted Mass Spec Environment (v.4.2), R Project for Statistical Computing (v.3.5.1), Stringr String Manipulator in R (v.1.3.1), Readr Data Reader for R (v.1.1.1), Ggplot2 figure creator for R (v.3.1.0), Venn Diagram package in R (v.1.6.20), Plyr Data Manipulation package in R (v.1.8.4), Dplyr Data Manipulation in R (v.0.7.7), Python Programming Language (v.2.7.14), SciPy Statistical Functions in Python (v.1.16.0), StatsModels Statistical Methods in Python (v.0.9.0), bio3d for R (v.2.4-3), Proteome Discoverer (v.2.5).<br><br>The custom code used in the manuscript is explained in detail in the methods section so that it can be readily re-created by other groups. The code can be provided upon request.

For manuscripts utilizing custom algorithms or software that are central to the research but not yet described in published literature, software must be made available to editors and reviewers. We strongly encourage code deposition in a community repository (e.g. GitHub). See the Nature Research guidelines for submitting code & software for further information.

Data

Policy information about availability of data

All manuscripts must include a data availability statement. This statement should provide the following information, where applicable:

- Accession codes, unique identifiers, or web links for publicly available datasets
- A list of figures that have associated raw data
- A description of any restrictions on data availability

All data produced or analyzed for this study are included in the published article (and its Supplementary Information files) or are available from the corresponding

author upon reasonable request. Proteomics data have been deposited at ProteomeXchange via the PRIDE database (http://www.proteomexchange.org) and are publicly available as of the date of publication: Accession numbers PXD044625 and 10.6019/PXD044625 (https://proteomecentral.proteomexchange.org/cgi/GetDataset?ID=PXD044625). RNA-seq data are available on NCBI Gene Expression Omnibus, under accession number GSE240318 (https://www.ncbi.nlm.nih.gov/geo/query/acc.cgi?acc=GSE240318). The crystallographic data supporting this work are deposited at the Cambridge Crystallographic Datacenter (CCDC) under the CCDC deposition number 2272256. These data can be obtained free of charge from The CCDC via www.ccdc.cam.ac.uk/structures.

Field-specific reporting

Please select the one below that is the best fit for your research. If you are not sure, read the appropriate sections before making your selection.

☒ Life sciences ☐ Behavioural & social sciences ☐ Ecological, evolutionary & environmental sciences

For a reference copy of the document with all sections, see nature.com/documents/nr-reporting-summary-flat.pdf

Life sciences study design

All studies must disclose on these points even when the disclosure is negative.

Sample size	No statistical methods were used to predetermine sample size. Sample sizes were selected according to literature showing similar methods of analysis: doi: 10.1038/s41589-019-0404-5, doi: 10.1038/nchem.2826, doi: 10.1038/s41587-020-00778-3.
Data exclusions	No data were excluded.
Replication	All mass spectrometry, biochemistry, and cell biology studies were successfully replicated on different days to verify reproducibility of the experimental findings. In general, studies were performed to include 3 independent replicates. For certain datasets, up to 6 independent replicates were performed.
Randomization	All experiments were performed on mammalian cells grown under identical conditions so randomization was not applicable.
Blinding	All experiments were performed on mammalian cells grown under identical conditions so blinding was not applicable.

Reporting for specific materials, systems and methods

We require information from authors about some types of materials, experimental systems and methods used in many studies. Here, indicate whether each material, system or method listed is relevant to your study. If you are not sure if a list item applies to your research, read the appropriate section before selecting a response.

Materials & experimental systems

n/a	Involved in the study	
☐	☒	Antibodies
☐	☒	Eukaryotic cell lines
☒	☐	Palaeontology and archaeology
☒	☐	Animals and other organisms
☒	☐	Human research participants
☒	☐	Clinical data
☒	☐	Dual use research of concern

Methods

n/a	Involved in the study	
☒	☐	ChIP-seq
☒	☐	Flow cytometry
☒	☐	MRI-based neuroimaging

Antibodies

Antibodies used	ANTI-FLAG antibody produced in rabbit, Sigma Aldrich, Cat.#:F7425-2MG, Batch#:0000131574 Dilution: 1:1,000<br>ANTI-GAPDH antibody produced in rabbit, Cell Signaling Technology, Cat.#:2118, Lot#14 Dilution: 1:1000<br>Anti-rabbit antibody produced in goat DyLight 550 conjugated, invitrogen, Cat.#:84541 Lot#TC264353 Dilution: 1:10000
Validation	ANTI-FLAG antibody produced in rabbit, Sigma Aldrich, Cat.#:F7425, Validated against FLAG-tagged proteins per manufacturers website. GAPDH: GAPDH Rabbit mAb, CST #2118S, Validated against human GAPDH in Hela, HUVEC and 3T3 cells per manufacturer website:<br><br>CST (GAPDH, Rabbit, 1:1000, 2118S) - To ensure antibodies will work in experiments, CST adheres to the Hallmarks of Antibody Validation™, six complementary strategies that can be used to determine the functionality, specificity, and sensitivity of an antibody in any given assay. Validation methods include: 1) binary model, 2) ranged expression, 3) orthogonal data, 4) multiple antibodies, 5) heterologous expression, and 5) complementary assays. According to the manufacturer, this product is approved for west blotting.<br><br>Millipore-Sigma (FLAG, 1:1000, F7425) - The standard antibody validation processes include verification for each recommended immunodetection application. Each of the thousands of antibodies in the Millipore-Sigma portfolio are certified through their standard validation process to ensure quality and reproducibility. This product was validated using an E. coli periplasmic extract

expressing an N-terminal FLAG fusion protein.

Eukaryotic cell lines

Policy information about cell lines

Cell line source(s)	Jurkat, HEK293T, A549 and HeLa cells were purchased from ATCC, DM93 were originally created by Dr. Seigler at Duke University Medical Center, http://www.jimmunol.org/content/jimmunol/142/9/3329. fu11.pdf
Authentication	None of the cell lines used were authenticated.
Mycoplasma contamination	Cell lines were not tested for mycoplasma contamination
Commonly misidentified lines (See ICLAC register)	Commonly misidentified cell lines were not used in this study.
